# A design‐by‐treatment interaction model for network meta‐analysis and meta‐regression with integrated nested Laplace approximations

**DOI:** 10.1002/jrsm.1285

**Published:** 2018-01-16

**Authors:** Burak Kürsad Günhan, Tim Friede, Leonhard Held

**Affiliations:** ^1^ Department of Medical Statistics University Medical Center Göttingen Göttingen Germany; ^2^ Epidemiology, Biostatistics and Prevention Institute University of Zurich Zurich Switzerland

**Keywords:** Bayesian inference, design‐by‐treatment interaction model, INLA, network meta‐analysis, network meta‐regression

## Abstract

Network meta‐analysis (NMA) is gaining popularity for comparing multiple treatments in a single analysis. Generalized linear mixed models provide a unifying framework for NMA, allow us to analyze datasets with dichotomous, continuous or count endpoints, and take into account multiarm trials, potential heterogeneity between trials and network inconsistency. To perform inference within such NMA models, the use of Bayesian methods is often advocated. The standard inference tool is Markov chain Monte Carlo (MCMC), which is computationally expensive and requires convergence diagnostics. A deterministic approach to do fully Bayesian inference for latent Gaussian models can be achieved by integrated nested Laplace approximations (INLA), which is a fast and accurate alternative to MCMC. We show how NMA models fit in the class of latent Gaussian models and how NMA models are implemented using INLA and demonstrate that the estimates obtained by INLA are in close agreement with the ones obtained by MCMC. Specifically, we emphasize the design‐by‐treatment interaction model with random inconsistency parameters (also known as the Jackson model). Also, we have proposed a network meta‐regression model, which is constructed by incorporating trial‐level covariates to the Jackson model to explain possible sources of heterogeneity and/or inconsistency in the network. A publicly available R package, nmaINLA, is developed to automate the INLA implementation of NMA models, which are considered in this paper. Three applications illustrate the use of INLA for a NMA.

## 1 INTRODUCTION

Network meta‐analysis (NMA)^1^ or mixed treatment comparison,^2^ which is a generalization of the pairwise (2 treatments) meta‐analysis,^3^ allows us to compare multiple treatments, although they have not been evaluated directly in a single trial. In recent years, with the increasing number of alternative treatment options, NMA gains an increasing popularity especially in the medical literature.^4^ The NMA can be regarded as the next generation evidence synthesis tool or the new norm for comparative effectiveness research.^5^

Many statistical models and parametrizations have been proposed for NMA. The standard approach to NMA is the *contrast‐based* model where the information of the relative treatment effects, expressed for example as log odds ratios, is pooled over trials. An alternative approach is the *arm‐based* (AB) model^6^ where absolute effects on each treatment arm, for instance, log odds, are pooled. For further details of these  2 methods, we refer readers to Piepho,^7^ Dias, and Ades^8^ and Hong et al.^9^ In this paper, we exclusively consider contrast‐based  modeling approaches, and we return to  AB models in the discussion.

A contrast‐based model can be defined using a *difference‐based likelihood* or an *arm‐based likelihood* (not to be confused with  AB models).^8^ The difference‐based likelihood approach uses a normal approximation to produce a summary estimate and its variance for each relative treatment effect. Also, if there are no events in at least one of the  trial arms for datasets with dichotomous endpoints, a continuity correction is needed. On the other hand, a contrast‐based model with an arm‐based likelihood avoids normal approximations and continuity corrections  and uses, for instance, a Binomial likelihood for datasets with dichotomous  end points. For these reasons, an arm‐based likelihood approach is preferable , and hence, we focus on these  types of models in the following.

Two of the most important challenges regarding NMA models are heterogeneity between trials and lack of consistency (inconsistency) in estimated treatment effects. Inconsistency arises when treatment effects obtained by direct evidence and indirect evidence(s) do not agree. The NMA models may be divided into 2 categories according to their approaches to inconsistency. Firstly, the *loop‐inconsistency* approach assumes that an inconsistency only occurs in closed loops of the network; these are represented by inconsistency random effects^2^ or  node splitting.^10^ Secondly, the *design‐inconsistency* approach was introduced to handle issues of the loop‐inconsistency method with the presence of  multiarm trials. The inconsistency parameters are treated as fixed effects by Higgins et al^11^ and as random effects by Jackson et al^12^ we refer the model using random inconsistency parameters as Jackson model. Moreover, to explain possible sources of heterogeneity and inconsistency in the network, a network meta‐regression model (an extension of  an NMA model by including study‐level covariates) can be used.^13^

The NMA models can be treated using frequentist methods. Recently,  to fit the Jackson model with a difference‐based likelihood, Jackson et al^14^ and Jackson et al^15^ proposed  2 estimation methods, which are extensions of the pairwise random‐effects meta‐analysis introduced by DerSimonian and Laird.^16^ Furthermore, a likelihood‐based method was introduced by Law et al^17^ and a Paule‐Mandel estimator suggested by Jackson et al^18^ to fit the Jackson model with a difference‐based likelihood. Alternatively, Bayesian inference is often used to fit NMA models. The standard way for a Bayesian inference is Markov chain Monte Carlo (MCMC), which is a simulation‐based technique. The statistical software packages WinBUGS,^19^ OpenBUGS,^20^ JAGS,^21^ and Stan 22 are popular  MCMC tools. However, MCMC is computationally expensive and requires the careful inspection of convergence diagnostics by the user. A deterministic approach to do Bayesian inference for latent Gaussian models (LGMs) has been proposed by Rue et al,^23^ the integrated nested Laplace approximations (INLA), which is a fast and accurate alternative to MCMC. Paul et al^24^ introduced INLA implementation of bivariate meta‐analyses of diagnostic test studies. Sauter and Held^25^ showed that many NMA models that are in the class of LGMs and INLA can be used as an inference technique alternative to MCMC for NMA models. They demonstrated how INLA can be applied to a NMA model with difference‐based likelihood,^1^ with arm‐based likelihood^2^ and the node‐splitting approach.^10^

The primary contribution of this paper is to introduce the usage of INLA for statistical inference within the Jackson model with arm‐based likelihood. Moreover, we propose a network meta‐regression model as an extension of the Jackson model. We use a common regression framework, which allows us to analyze datasets with different type of outcomes including continuous, dichotomous, and count using INLA. Another contribution to the existing literature is the introduction of an R package, nmaINLA(https://CRAN.R‐project.org/package=nmaINLA), which is developed to automate INLA implementation of NMA models described in the paper and publicly available from CRAN. We demonstrate that the estimates obtained by INLA are very close to the ones by MCMC. In Section 2, we describe different NMA models including methods to deal with the inconsistency in the network. In Section 3, we discuss Bayesian inference of NMA models using INLA. The INLA implementation of NMA models is demonstrated using  3 applications in Section 4. We close with some conclusions and provide a brief discussion.

**Highlights**

**What is already known**: Bayesian inference using Markov chain Monte Carlo (MCMC) is one of the most popular approaches for fitting network meta‐analysis (NMA) models to take into account possible heterogeneity and inconsistency in the network.

**What is new**: As an alternative to MCMC, integrated nested Laplace  approximations (INLA) can be used to make inference for widely used NMA models including the design‐by‐treatment interaction model. INLA is faster than MCMC and does not require checking of convergence diagnostics unlike MCMC. Furthermore, a new network meta‐regression model is suggested to explain possible sources of heterogeneity and/or inconsistency.

**Potential impact for RSM readers outside the authors' field**: To make it more accessible to NMA‐practitioners, a publicly available R package, nmaINLA, is developed for fitting the discussed NMA models using INLA.

## 2 STATISTICAL MODELS FOR NMA

We use a generalized linear mixed model (GLMM) framework to describe network meta‐analysis models. In Section 2.1, we describe a fixed effect model, then a consistency model in Section 2.2, and continue with the design‐inconsistency in Section 2.3. We propose a novel network meta‐regression model in Section 2.4.

### 2.1 Fixed effect model

The models described here follow the ones described in Dias et al^26^ and Dias and Ades.^8^ To model datasets with different type of  end points, we describe a common regression framework. The essential idea is that the basic model remains the same, but the likelihood and the link function can change to reflect the nature of the data (continuous, dichotomous, or count), and the sampling process that generated it (eg, Normal, Binomial, or Poisson). As a special case, a pairwise meta‐analysis is just a network meta‐analysis with only 2 treatments included in the network.

For simplicity, we firstly consider a fixed effect NMA model. Each trial *i*∈{1,2,…,*S*} has treatment arms, which are defined using trial‐specific treatment indices *k*∈{1,2,…,*K*} where *K*≥2, and *T* is the total number of treatments. The first treatment of a trial *i* is baseline treatment, *t*_1_, compared with the  nonbaseline treatments. We distinguish trial‐specific baseline treatment *t*_1_ from the *reference treatment*, say treatment 1, which may or may not be present in trial *i*. For instance, to parametrize  an NMA dataset, if there exists placebo treatment in one of the trials, placebo can be chosen as the reference treatment, but placebo do not necessarily present in each trial in the dataset. We specify a likelihood with some unknown parameters, $p(y|\theta_{i,t_{k}})$, where *y* is the observed data and $\theta_{i,t_{k}}$ is the relative treatment effect parameter of interest of arm *t*_*k*_ of study *i*. A link function *G*(.) is used to transform $\theta_{i,t_{k}}$ onto a scale where its effects can be assumed to be additive

(1) $$G(\theta_{i,t_{k}})=\left\{\begin{array}{cc} \mu_{it_{1}}, & \text{if}\;k=1\\ \mu_{it_{1}}+d_{t_{1}t_{k}}, & \text{otherwise,} \end{array}\;\right.$$

where $\mu_{it_{1}}$ is the absolute treatment effect of baseline treatment (*t*_1_) in trial *i* and it is treated as a nuisance parameter. Hereafter, subscript *t*_1_ is dropped from $\mu_{it_{1}}$, since it is redundant. The main interest is in $d_{t_{1}t_{k}}$, which is the relative treatment effect between *t*_1_ and *t*_*k*_.

The parametrization of the network is achieved using *basic parameters*, which can be chosen as any set including *T*−1 available treatment comparisons in the network. All other available treatment comparisons in the network are *functional parameters*, which can be calculated from the basic parameters. For instance, if $d_{t_{1}t_{2}}$ and $d_{t_{1}t_{3}}$ are basic parameters in the network, then a functional parameter $d_{t_{2}t_{3}}$ can be calculated using following equation

(2) $$d_{t_{2}t_{3}}=d_{t_{1}t_{3}}-d_{t_{1}t_{2}}.$$

Now, we consider the models for datasets with a dichotomous outcome. Assume that the number of events $y_{i,t_{k}}$ and the number of patients $n_{i,t_{k}}$ are given in treatment arm *t*_*k*_ of trial *i*. Then  the likelihood function can be written as $y_{i,t_{k}}∼\text{Bin}(\pi_{i,t_{k}},n_{i,t_{k}})$ and a logit link function is used to define the treatment effect parameter $\left(G(\theta_{i,t_{k}})=\text{logit}(\theta_{i,t_{k}}\right)$ in Equation 1).

Likewise, the NMA models for continuous outcome data can be formulated using a normal likelihood with identity link function.^26^ When the data available for the NMA are counts, a Poisson likelihood with log link can be used.^26^ For simplicity, from this point on, we exclusively consider models for dichotomous outcomes.

### 2.2 Consistency model

As for the pairwise meta‐analysis, heterogeneity between trials can be taken into account using random effects in the NMA context. If there is no  multiarm trial in the network, trial‐specific heterogeneity random effects, say $\gamma_{i,t_{1}t_{k}}$'s, are added to Equation 1 and it is assumed that the $\gamma_{i,t_{1}t_{k}}$'s are independently (and normally) distributed with mean zero and some variance (heterogeneity variance). However, treatment comparisons in a  multiarm trial are not independent, since all  nonbaseline treatments are compared to the same baseline treatment. To illustrate this situation, consider a  3‐arm trial *i* including treatments *t*_1_, *t*_2_, and *t*_3_.  To account for this dependency within trial *i*, a multivariate normal distribution with mean zero vector is used for the random effects vector $\gamma_{i}={\left(\gamma_{i,t_{1}t_{2}},\gamma_{i,t_{1}t_{3}}\right)}^{⊺}$. A simple but a convenient structure of the covariance matrix of the multivariate normal distribution is suggested by Higgins and Whitehead.^27^ In general, we model heterogeneity as 

(3) $$\gamma_{i}∼N_{T-1}(0,\Sigma_{\gamma }),\;$$

where **Σ**_*γ*_ is a symmetric homogeneous covariance matrix with diagonal entries equal to *τ* ^2^ and  nondiagonal entries set to *τ* ^2^/2. This form of covariance matrix is justified by the assumption that heterogeneity variances are the same for each treatment comparison for each trial.^27^ Inclusion of heterogeneity random effects in Equation 1 with a logit link leads to the following model

(4) $$\text{logit}(\pi_{i,t_{k}})=\left\{\begin{array}{cc} \mu_{i}, & \text{if}\;k=1\\ \mu_{i}+d_{t_{1}t_{k}}+\gamma_{i,t_{1}t_{k}}, & \text{otherwise}. \end{array}\right.$$

We refer to this model as *consistency model*, since it assumes that there is no inconsistency in the network.

### 2.3 Design‐inconsistency model

The consistency assumption may not hold up if there is discrepancy between evidence coming from direct estimates and indirect estimates.  To take into account the possible inconsistency in the network, the Lu‐Ades model^2^ or design‐inconsistency approaches can be used. The Lu‐Ades model adds inconsistency parameters to closed loops where loop inconsistency may arise. However, Higgins et al^11^ showed that the estimates of the Lu‐Ades model depend on treatment ordering. Also, Jackson et al^28^ have shown that the Lu‐Ades model is a restricted version of the design‐inconsistency model. Therefore, we exclusively consider the design‐inconsistency model in this paper. *The design‐by‐treatment interaction model* for inconsistency was introduced in Higgins et al.^11^ The central concept of this approach is the *design*, which refers to the sets of treatments included in a particular study. We use $D(i)=1,2,\text{⋯},\bar{D}$ to denote the design of trial *i*. For example, if the first design compares treatments 1 and 2, then *D*=1 refers to  2 arm‐trials, which compare treatments 1 and 2.  Design inconsistency means differences in treatment effects between designs. Higgins et al^11^ treated  design‐inconsistency parameters as fixed effects, whereas Jackson et al^12^ treated them as random effects; hereafter, we use the term *Jackson model* for the latter. The advantage of treating inconsistency parameters as fixed effects is that no common distribution assumption is needed as in the Jackson model.^11^ On the other hand, the Jackson model introduces inconsistency parameters as random effects. Hence, inconsistency is treated as an additional source of variation alongside heterogeneity as in the consistency model. The Jackson model also facilitates the sensitivity analyses in terms of a single sensitivity parameter (the inconsistency variance), and more importantly, we can “estimate average treatment effects across all designs, rather than the design‐specific treatments effects we obtain when using fixed effects for the inconsistency parameters .”^12^ In this section, we only discuss the Jackson model.

The Jackson model relaxes the consistency relation $d_{t_{1}t_{k}}=d_{t_{l}t_{k}}-d_{t_{l}t_{1}}$ to $d_{t_{1}t_{k}}=d_{t_{l}t_{k}}-d_{t_{l}t_{1}}+\omega_{t_{1}t_{k}}^{D(i)}$ where $\omega_{t_{1}t_{k}}^{D(i)}$ is a design‐specific inconsistency random effect. Hence, $\omega_{t_{1}t_{k}}^{D(i)}$ is added to Equation 4 resulting in

(5) $$\text{logit}(\pi_{i,t_{k}})=\left\{\begin{array}{cc} \mu_{i}, & \text{if}\;k=1\\ \mu_{i}+d_{t_{1}t_{k}}+\gamma_{i,t_{1}t_{k}}+\omega_{t_{1}t_{k}}^{D(i)}, & \text{otherwise}. \end{array}\right.$$

For the inconsistency random effects, Jackson et al^12^ proposed similar assumptions for the heterogeneity random effects (see Equation 3):

(6) $$\omega^{D(i)}∼N_{T-1}(0,\Sigma_{\omega }),\;$$

where **Σ**_*ω*_ denotes a square matrix with the diagonal entries that are all *κ* ^2^ and all other entries are *κ* ^2^/2. The structure **Σ**_*ω*_ is justified by assuming that the inconsistency variances across designs  are same for every treatment comparisons. The inconsistency variance *κ* ^2^ is a measure of the degree of the inconsistency in the whole network, and each inconsistency random effect $\omega_{t_{1}t_{k}}^{D(i)}$ describes where particular inconsistencies arise. Note that inconsistency random effects are defined based on the data at hand, and they should be specified after the set of the designs, *D*(*i*)^*′*^ *s*, of the network is determined. To illustrate how to choose the inconsistency parameters, consider a simple NMA dataset  that includes one 3‐arm trial and two  2‐arm trials. The  3‐arm trial includes treatments 1, 2, 3; and one  2‐arm trial includes treatments 1  and 2, and the other trial includes 1 and 3. In this example, there are  3 different designs (*D*=1,2,3) and 4 different inconsistency random effects, namely, $\omega_{12}^{1},\omega_{13}^{1},\omega_{12}^{2}$, and $\omega_{13}^{3}$.

To parametrize the network, any *T*−1 treatment comparisons can be chosen as basic parameters as in the fixed effect and consistency model. However, for the implementation of the Jackson model (as well as fixed effect and consistency models), as described in Law et al,^17^ we determine a reference treatment, treatment 1, and then choose relative treatment effects compared to the reference treatment as basic parameters $d_{1t_{k}}$'s where *t*_*k*_≠1. This is done only to make implementation and interpretation easier, since the Jackson model is invariant to the choice of basic parameters.

### 2.4 Network meta‐regression

The exploration of *covariate‐by‐treatment interactions* in  an NMA context or *network meta‐regression* 13 is used to explain potential sources of heterogeneity in the network. These models can be constructed by extending the consistency or the fixed effect model with the inclusion of study‐level covariates, say *x*_*i*_'s. Therefore,  an NMA model is a network meta‐regression model without covariates. On the other hand, an investigation of covariates  to explain inconsistency in the network may also be of interest. To achieve this, some relevant covariates can be incorporated to Jackson model. As a result, if the inconsistency variance is substantially decreased, then we may conclude that the included covariate explained the reduced amount of the total inconsistency in the network.^12^ We therefore propose a network meta‐regression model to achieve this. On the other hand, even if we only include randomized controlled trials for our analysis, meta‐regression (pairwise or network) inherits the challenges attached to all observational studies, for example, confounding. In other words, we may obtain a correlation between a covariate and a relative treatment effect; however, the correlation may not be a causation. This is because it is not possible to randomize patients to one covariate (see Thompson and Higgins,^29^, section 3 for further discussion of limitations of meta‐regression).

As explained in Dias et al,^13^ covariate‐by‐treatment interactions can be  modeled in 3 different ways: unrelated interaction coefficients for each treatment, exchangeable and related interaction coefficients, and one constant interaction coefficient for all treatments. By following the suggestion of Dias et al,^13^ we only discuss the third model because of its easier interpretation. The third model assumes that all covariate‐by‐treatment interactions are identical; that is, a constant interaction coefficient *β* is assumed across all treatments relative to the reference treatment implying the same covariate effect for each treatment relative to the reference ( $\beta =\beta_{1t_{k}}$ for any *t*_*k*_≠1).^30^ That means, the treatment effects relative to the reference treatment, *d*_12_,*d*_13_,…,*d*_1*T*_, now become *d*_12_+*x*_*i*_·*β*,*d*_13_+*x*_*i*_·*β*,…,*d*_1*T*_+*x*_*i*_·*β*. In the case of continuous covariates, we use centered covariate values $\left(x_{i}-\bar{x}\right)$ because this is computationally more stable. To fit the proposed network meta‐regression model, Equation 5 becomes

(7) $$\text{logit}(\pi_{i,t_{k}})\;=\;\left\{\;\begin{array}{cc} \mu_{i}, & \text{if}\;t_{k}=1\\ \mu_{i}+\;d_{1t_{k}}\;+\;\gamma_{i,1t_{k}}\;+\;\omega_{1t_{k}}^{D(i)}\;+\;(x_{i}-\bar{x})\cdot \beta , & \text{otherwise,} \end{array}\;\right.$$

where *β* represents the log odds ratio of an event per unit change in the (centered) covariate for treatment *t*_*k*_ relative to the reference treatment 1. Note that, with this model, covariate‐by‐treatment interactions of a treatment effect relative to a treatment other than the reference treatment is assumed to be zero. For example, if we consider covariate‐by‐treatment interaction of treatment 3 relative to treatment 2, then interaction terms cancel out as follows:

(8) $$(d_{13}+(x_{i}-\bar{x})\cdot \beta )-(d_{12}+(x_{i}-\bar{x})\cdot \beta )=d_{13}-d_{12}.$$

This suggests that the choice of the reference treatment is important, and it affects the interpretation of the results. Therefore, we need to determine a reference treatment (say treatment 1), and basic parameters as $d_{1t_{k}}$'s where *t*_*k*_≠1. Otherwise, we do not obtain any meaningful interpretation from the results of the fitted model.

## 3 BAYESIAN INFERENCE FOR FITTING NETWORK META‐ANALYSIS MODELS USING INLA

 The NMA models that we described in Section 2 are in the class of  GLMMs. Fong et al^31^ have shown that INLA can be used to fit GLMMs; hence, it is possible to make inference for our NMA models using INLA. To be more precise, we can show that NMA models are in the family of  LGMs as described in Rue et al.^32^, section 2.1 This can be achieved by describing NMA models with a  3‐stage model formulation as follows:

**Stage 1**: Assume that *N* is the total number of arms of all trials, ***μ*** is the vector of all baseline treatment effects, ***d***_*b*_ is the vector of the basic parameters, and *β* is the constant interaction coefficient. Also, the random effects vector ***γ*** contains all trial‐specific heterogeneity random effects. Likewise, ***ω*** contains all inconsistency random effects, $\omega_{t_{1}t_{k}}^{D(i)}$ for the Jackson model. The observed data ***y***={*y*_1_,…,*y*_*N*_} is described by the likelihood
  (9) $$p(y|\alpha ,\Psi )=\prod_{i=1}^{N}p(y_{i}|\alpha_{i},\Psi ),\;$$
  where ***α***=(***μ***,***d***_*b*_,*β*,***γ***,***ω***) includes all model parameters, and hyperparameters are denoted by **Ψ**=(Ψ_1_=*τ* ^2^,Ψ_2_=*κ* ^2^).
**Stage 2**: It is assumed that random effects ***γ*** and ***ω*** are both normally distributed (Equations 3 and 6). Also, if we assume normal priors for all elements of ***μ***, ***d***_*b*_, and for *β*, then the joint distribution for ***α*** has a multivariate normal distribution $\left(\alpha ∼N(0,\Sigma_{\Psi })\right)$, which is called the latent Gaussian field.
**Stage 3**: Lastly, priors are defined for the hyperparameters **Ψ**=(Ψ_1_=*τ* ^2^,Ψ_2_=*κ* ^2^). Note that normal as well as  nonnormal priors can be selected for the hyperparameters.

Now, we briefly review how INLA computation approach works. Basically, the INLA methodology uses multiple Laplace approximations^33^ and numerical integration. For our NMA models, the joint posterior distribution is given by

$$\begin{array}{ccc} p(\alpha ,\Psi |y) & \propto p(\Psi )\times p(\alpha |\Psi )\times \prod_{i=1}^{N}p(y_{i}|\alpha_{i},\Psi ) & \\ & \propto p(\Psi )\times {\Sigma_{\Psi }}^{-1/2}\exp\left(-\frac{1}{2}\alpha^{T}{\Sigma_{\Psi }}^{-1}\alpha +\sum_{i=1}^{N}\log\left(p(y_{i}|\alpha_{i},\Psi )\right)\right). \end{array}$$

Our main objective is computing the marginal posterior distributions of ***α*** and **Ψ**. For ***α***, we can write

(10) $$p(\alpha_{i}|y)=\int p(\alpha_{i}|\Psi ,y)p(\Psi |y)d\Psi ,\;$$

which is evaluated via the approximation

(11) $$\tilde{p}(\alpha_{i}|y)=\int \tilde{p}(\alpha_{i}|\Psi ,y)\tilde{p}(\Psi |y)d\Psi .$$

Thus, we need to calculate $\tilde{p}(\Psi |y)$ and $\tilde{p}(\alpha_{i}|\Psi ,y)$. For $\tilde{p}(\Psi |y)$, we can write  

$$\begin{array}{ccc} p(\Psi |y) & =\frac{p(\alpha ,\Psi |y)}{p(\alpha |\Psi ,y)} & \\ & =\frac{p(y|\alpha ,\Psi )p(\alpha ,\Psi )p(\Psi )}{p(y)}\frac{1}{p(\alpha |\Psi ,y)}\\ & \approx \frac{p(y|\alpha ,\Psi )p(\alpha |\Psi )p(\Psi )}{\tilde{p}(\alpha |\Psi ,y)}{|}_{\alpha =\alpha^{*}(\Psi )}=\tilde{p}(\Psi |y), \end{array}$$

where $\tilde{p}(\alpha |\Psi ,y)$ is the Laplace approximation of *p*(***α***|**Ψ**,***y***) and ***α*** ^∗^(**Ψ**) is the mode for a given **Ψ**. The calculation of $\tilde{p}(\alpha_{i}|\Psi ,y)$ is performed using the simplified Laplace approximation, which is based on a Taylor expansion of the Laplace approximation around mode.^34^

Then  Equation 11 can be solved using numerical integration:

(12) $$\tilde{p}(\alpha_{i}|y)\approx \sum_{j}\tilde{p}(\alpha_{i}|\Psi^{j},y)\tilde{p}(\Psi^{j}|,y)\Delta_{j}$$

for some integration points **Ψ** ^*j*^ with appropriate weights **Δ**_***j***_. The weights **Δ**_***j***_ depend on the selection of the values **Ψ** ^*j*^. The default selection scheme in INLA is the central composite design strategy. In the  central composite design strategy, the mode of Ψ^∗^ of $\tilde{p}(\Psi^{j}|,y)$ and the Hessian at the mode are located. Then  some relevant points in **Ψ**‐space (a *q*‐dimensional space where *q* is the number of hyperparameters) are selected for performing second‐order approximation (see  Rue et al^23^, section 6.5 for details). Therefore, with this strategy, instead of laying out a dense grid of integration points , for example, using points with equal weights, only a limited number of well‐chosen points are used. Lastly, marginal posterior densities for *p*(Ψ_*k*_|***y***) can be obtained similarly from *p*(**Ψ**|***y***).^35^

The INLA R package, hereafter referred as R‐INLA, provides an interface for R to INLA (a free‐standing program) so that models can be fitted using standard R commands. Additional to posterior marginals, R‐INLA also provides estimates of the deviance information criterion (DIC),^36^ and the Watanabe‐Akaike information criterion.^37^ The R‐INLA package is available on INLA website (http://www.r‐inla.org/). The use of R‐INLA to fit different NMA models including Lu‐Ades model is explained in Sauter and Held^25^ and the accompanying  Supporting Information. However, for the practitioner carrying out  an NMA, the range of options and the required knowledge of available features in R‐INLA might be overwhelming. Fortunately, the data preparation and  postprocessing steps can be automated. To this end, we present a new R package nmaINLA, which is a purpose‐built package defined on top of R‐INLA extracting only the features needed for network meta‐analysis. Our package nmaINLA(https://CRAN.R‐project.org/package=nmaINLA) implements all NMA models described in the text. nmaINLA extracts the features needed for NMA models from R‐INLA and presents in an intuitive way. Therefore, users do not need to know the structure of the general R‐INLA output object. A tutorial for the installation and how to use the nmaINLA package is given in the  Supporting Information. The development version of nmaINLA is available on Github (https://github.com/gunhanb/nmaINLA). For the NMA models  that are not supported by nmaINLA, one may extend our package or use directly the R‐INLA.

We compare the results obtained using the INLA approach with MCMC. For the MCMC method, we use JAGS 21 from within R with the help of R2jags 38 R package. Raw data, R code, and JAGS code to reproduce all results of this paper are presented in the  Supporting Information. All analyses were run on a laptop with Intel(R) Core(TM) 4 Duo i3‐6100U processor 2.30  GHz.

## 4 APPLICATIONS

In this section, we illustrate INLA technique using  3 different applications. In Section 4.1,  an NMA dataset in Diabetes is considered as an example to evaluate the relative effect on HbA1c change of adding different oral glucose‐lowering agents. In Section 4.2, we analyzed  an NMA dataset comparing different interventions to aid smoking cessation. Lastly, a dataset is considered to compare number of treatments to prevent stroke in patients with atrial fibrillation in Section 4.3. For the prior specifications, we use independent normal priors with mean zero and variance 1000 for all components of ***α*** in all  3 applications. For the hyperparameters *τ* and *κ*, uniform priors on the interval [0, 5] were used in the first and second applications as in Jackson et al.^12^ In the third application, we used uniform priors on the interval [0, 2] for hyperparameters *τ* and *κ*, which we take from Batson et al.^39^ For implementations in JAGS, we used the BUGS code from the code given in Dias et al,^26^ Jackson et al,^12^ and Dias et al^13^ in  Sections  4.1,  4.2, and 4.3, respectively. Both for the Diabetes and Smoking applications, after burn‐in of 30 000 iterations, 20 000 iterations in the fixed effect and consistency models  and 50 000 iterations in the Jackson model were used to obtain posterior distributions. For the Stroke prevention application, after burn‐in of 50   000 iterations, 50 000 additional iterations were used in the fixed effect and consistency models (with and without covariate), and 100 000 additional iterations were used for the Jackson models (with and without covariate). For all  3 applications, 3 MCMC chains were used with 5 as the thinning parameter. The number of iterations was chosen to ensure that all Monte Carlo standard errors were around 0.005. Convergence diagnostics was checked using JAGS implementation of Gelman‐Rubin statistics,^40^ as well as visual inspection of traceplots and autocorrelation plots. We used DIC as a model comparison criterion, which is available from R‐INLA.

### 4.1 Diabetes: NMA with continuous endpoints

**Figure 1:**
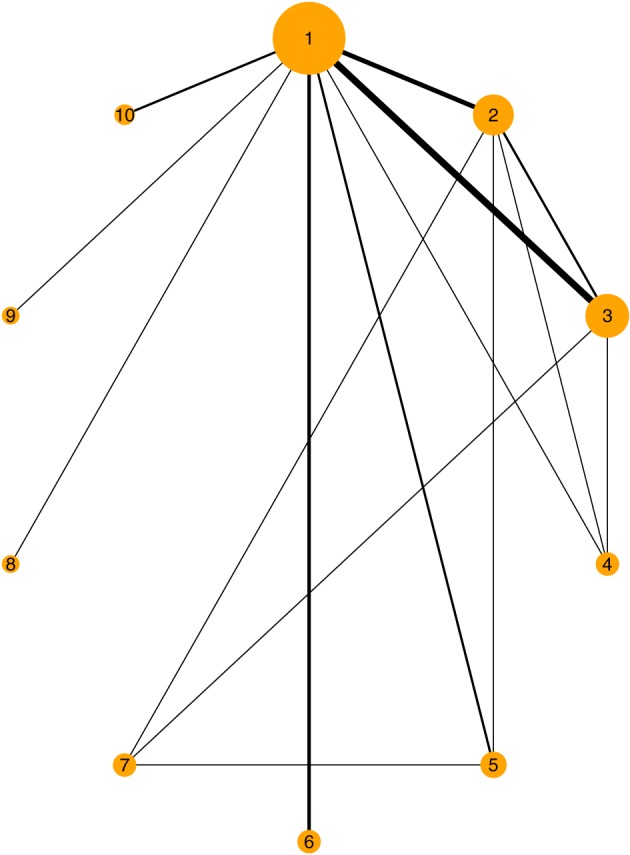
Network of trials of Diabetes application. Lines indicate that there is data available from one or more studies comparing the two treatments. Width of lines is proportional to the number of trials for that comparison. The size of the circle is proportional to the number of participants to that treatment [Colour figure can be viewed at http://wileyonlinelibrary.com]

The Diabetes dataset is originally analyzed by Senn et al,^41^ and the raw data are shown in  table AI of Senn et al.^41^ The dataset includes the results from 26 randomized controlled trials examining the effectiveness of adding various oral glucose‐lowering agents to a baseline sulfonylurea therapy in patients with type 2 diabetes. The outcome measured in the studies was either the mean HbA1c level at follow‐up or the mean change in HbA1c level from baseline to follow‐up. A total of 10 different treatment types were examined in these studies (1, placebo; 2, metformin; 3, rosiglitazone;  4, pioglitazone; 5, acarbose; 6, miglitol; 7, sulfonylurea alone; 8, sitagliptin; 9, vildagliptin; 10, benfluorex). Figure 1 shows the plot of the network of this dataset. One study included  3 treatment arms, while the rest of the studies included  2 treatment arms. There are 16 different designs in the network. The available data are the sample mean, standard deviation, and number of patients for each arm of each study. Firstly, we calculated each standard error of the sample mean. Since there is a continuous outcome available, a normal likelihood with identity link is used to fit the models for this application as described in Section 2.1. We have fitted the fixed effect model, the consistency model, and the Jackson model using both MCMC and INLA. To give some sense of how our nmaINLA package looks like in a routine data analysis, below we show the corresponding R code to fit the Jackson model

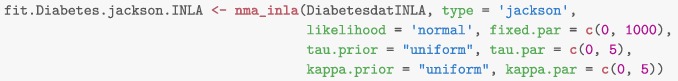

**Figure 2:**
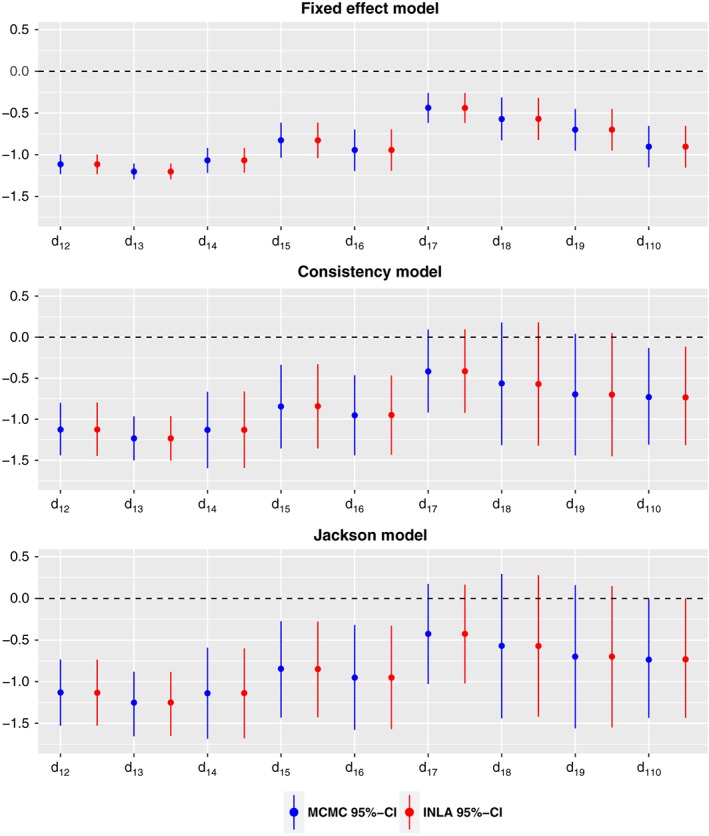
Median and 95% equi‐tailed credible interval (CI) of the marginal posterior distributions of all relative treatment effects by MCMC and by INLA for the Diabetes data

Figure 2 shows the posterior median and the 95% equi‐tailed credible interval (CI) obtained by INLA and by MCMC for all basic parameter estimates of the  3 fitted models. The estimates of heterogeneity and inconsistency standard deviations are displayed in Table 1. Individual inconsistency random effects are displayed in Table 2. Senn et al^41^ have fitted fixed effect and consistency models using frequentist methods to analyze the Diabetes data. Our results are in broad agreement with their results (see figures 5 and 7 in Senn et al^41^).

**Table 1:** Estimates of heterogeneity and inconsistency standard deviation of consistency and Jackson model for the Diabetes application

	**Consistency**		**Jackson**	
	MCMC	INLA	MCMC	INLA
**Heterogeneity (*τ*)**				
Posterior median	0.336	0.335	0.339	0.339
Lower b (95% CI)	0.217	0.218	0.216	0.216
Upper b (95% CI)	0.531	0.531	0.548	0.547
**Inconsistency (*κ*)**				
Posterior median			0.122	0.122
Lower b (95% CI)			0.006	0.007
Upper b (95% CI)			0.480	0.488

Abbreviations: INLA, integrated nested Laplace approximations; MCMC, Markov chain Monte Carlo.

**Table 2:** Estimated inconsistency parameters obtained from the fitted Jackson model for the Diabetes dataset

		**MCMC**		**INLA**	
**Design**	**Parameter**	Mean	Stdev	Mean	Stdev
1	$\omega_{1,2}^{1}$	−0.01	0.16	−0.01	0.15
2	$\omega_{1,5}^{2}$	−0.01	0.18	−0.01	0.17
	$\omega_{1,2}^{2}$	−0.00	0.18	−0.00	0.18
3	$\omega_{1,3}^{3}$	0.04	0.16	0.04	0.16
4	$\omega_{1,4}^{4}$	−0.02	0.17	−0.02	0.17
5	$\omega_{2,4}^{5}$	0.03	0.18	0.03	0.17
6	$\omega_{3,4}^{6}$	−0.01	0.17	−0.00	0.17
7	$\omega_{2,3}^{7}$	0.00	0.17	−0.00	0.16
8	$\omega_{3,7}^{8}$	0.06	0.19	0.06	0.18
9	$\omega_{5,7}^{9}$	−0.00	0.18	−0.00	0.17
10	$\omega_{1,5}^{10}$	0.01	0.18	0.01	0.17
11	$\omega_{1,8}^{11}$	−0.00	0.20	−0.00	0.19
12	$\omega_{1,9}^{12}$	−0.00	0.20	−0.00	0.19
13	$\omega_{2,7}^{13}$	−0.06	0.19	−0.05	0.18
14	$\omega_{1,6}^{14}$	0.00	0.20	−0.00	0.19
15	$\omega_{2,3}^{15}$	0.02	0.17	0.02	0.17
16	$\omega_{1,10}^{16}$	0.00	0.20	−0.00	0.19

Abbreviations: INLA, integrated nested Laplace approximations; MCMC, Markov chain Monte Carlo.

The median and 95% equi‐tailed  CI of the heterogeneity from Table 1 suggesting a substantial heterogeneity in the network. However, the estimates of the inconsistency are very close to zero, and also the individual inconsistency parameters from Table 2 are almost zero with high standard deviations. Therefore, we can conclude that there is no evidence of substantial inconsistency in the network. The DIC values of the fixed effect  model, the consistency model, and the Jackson model are 36.86, −28.82, and −28.27, respectively. The consistency model offers a large improvement in DIC compared to the fixed effect model, which confirms possible presence of the heterogeneity. The DIC value of the Jackson model is very close to the DIC value of the consistency model. However, as displayed in Figure 2, including inconsistency random effects has considerable impact on the credible intervals (hence, the precisions) of the basic parameters. Therefore, although there is not strong evidence of any inconsistency in this network, it has quite considerable impact when it is included in the model.

The MCMC and INLA methods gave very similar results. If we consider all  3 models, the largest absolute difference for the posterior median estimate based on MCMC and INLA among basic parameters was found in the Jackson model for *d*_18_(0.0059). Furthermore, the largest absolute difference of the INLA and MCMC posterior mean estimates of individual inconsistency random effects in the Jackson model was 0.0032. For the Jackson model, the MCMC run took 30  seconds while INLA only took 4.9  seconds.

### 4.2 Smoking cessation: NMA with dichotomous endpoints

**Figure 3:**
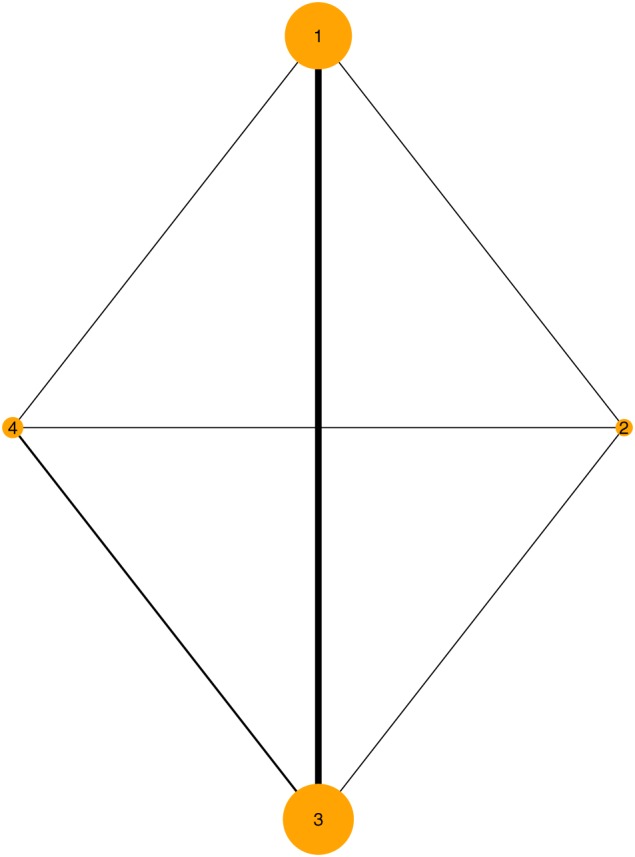
Network of trials of Smoking cessation [Colour figure can be viewed at http://wileyonlinelibrary.com]

The second application includes 24 trials investigating interventions to aid smoking cessation and has been considered by Jackson et al^12^ and Sauter and Held^25^ among others. The number of individuals who successfully quits smoking after 6 to 12 months is reported for  4 different interventions (1 , no contact; 2 , self‐help; 3 , individual counseling; and 4 , group counseling). The plot of the network is displayed in Figure 3. There are two  3‐arm trials, one for treatments 1, 3, and 4 and one for treatments 2, 3, and 4. And there are  8 different designs in the network.

**Figure 4:**
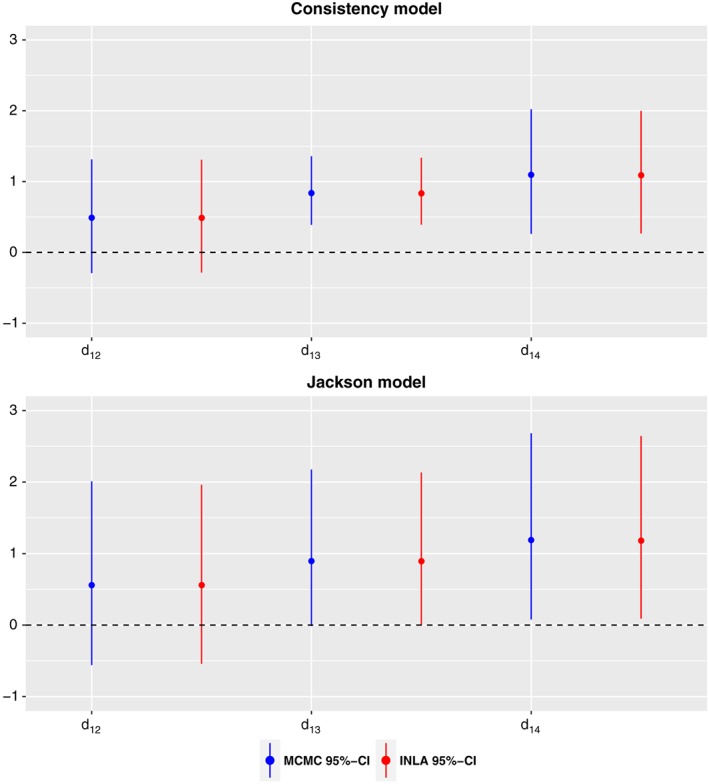
Median and 95% equi‐tailed credible interval (CI) of the marginal posterior distribution of all relative treatment effects by MCMC and by INLA for the Smoking cessation data

**Figure 5:**
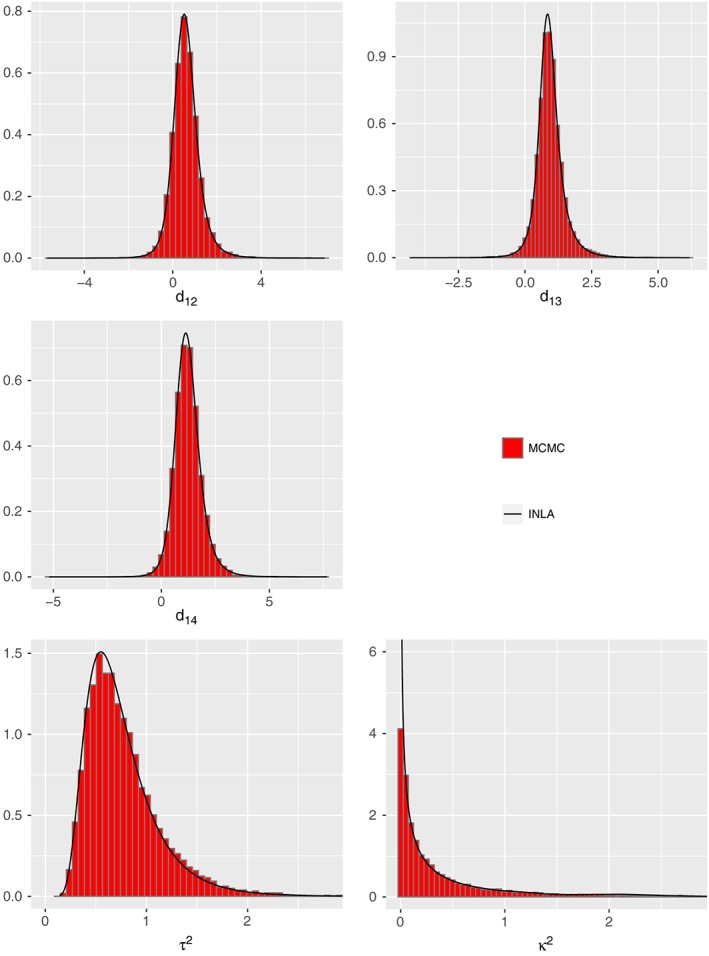
Marginal posterior density estimates of all basic parameters, the heterogeneity and inconsistency variances by MCMC (histogram) and by INLA (straight line) obtained from the fitted Jackson model for the Smoking cessation dataset [Colour figure can be viewed at http://wileyonlinelibrary.com]

Figure 4 shows the posterior median and the 95% equi‐tailed CI obtained by INLA and by MCMC for basic parameter estimates of the consistency and the Jackson model. Furthermore, the marginal posterior densities from the Jackson model are displayed in Figure 5 as histograms of the MCMC samples and as solid lines of the INLA results. Finally, Table 3 demonstrates the estimates of inconsistency random effects obtained by MCMC and INLA. Jackson et al^12^ presented the fitted consistency and Jackson model using MCMC for the Smoking dataset. We obtained very similar results with the results displayed in  table 3 of Jackson et al.^12^

**Table 3:** Estimated inconsistency parameters obtained from the fitted Jackson model for the Smoking dataset

		**MCMC**		**INLA**	
**Design**	**Parameter**	Mean	Stdev	Mean	Stdev
1	$\omega_{1,3}^{1}$	0.03	0.54	0.02	0.53
	$\omega_{1,4}^{1}$	−0.26	0.63	−0.28	0.64
2	$\omega_{2,3}^{2}$	−0.06	0.54	−0.07	0.55
	$\omega_{2,4}^{2}$	−0.09	0.54	−0.10	0.55
3	$\omega_{1,3}^{3}$	−0.08	0.50	−0.10	0.50
4	$\omega_{1,2}^{4}$	−0.12	0.56	−0.13	0.55
5	$\omega_{1,4}^{5}$	0.39	0.77	0.39	0.76
6	$\omega_{2,3}^{6}$	−0.10	0.54	−0.11	0.55
7	$\omega_{2,4}^{7}$	0.10	0.55	0.09	0.55
8	$\omega_{3,4}^{8}$	−0.04	0.51	−0.03	0.50

Abbreviations: INLA, integrated nested Laplace approximations; MCMC, Markov chain Monte Carlo.

The posterior median for the heterogeneity standard deviation is 0.82 with 95% CI [0.55, 1.3] suggesting that there may be notable heterogeneity in the network. The posterior median for the inconsistency standard deviation is 0.4 with 95% CI [0.02, 1.87], suggesting moderate but highly uncertain inconsistency. Moreover, when we examine the inconsistency random effects in Table 3, it is hard to claim that there is strong evidence for the inconsistency in this network, since standard deviations are very wide. The DIC values of the consistency model, and the Jackson model are 326.56  and 326.62, respectively. Since the DIC values are almost indistinguishable, we may conclude that no strong inconsistency in the network. On the other hand, as in the Diabetes application, including inconsistency parameters to the consistency model influences the precision of the basic parameters, which can be seen from Figure 4. This observation was also made by Jackson et al.^12^

We can conclude that both inference techniques, MCMC and INLA, give similar results in our analysis. Based on MCMC and INLA of the fitted Jackson model, the largest absolute difference for the posterior median estimate of the basic parameters was 0.0035 and for the posterior mean estimate of the inconsistency parameters was 0.017 $\left(\omega_{14}^{5}\right)$. For the Jackson model, the MCMC run took 34.2  seconds while the computing time was 6.5  seconds with INLA.

### 4.3 Stroke prevention:  network meta‐regression with dichotomous endpoints

**Figure 6:**
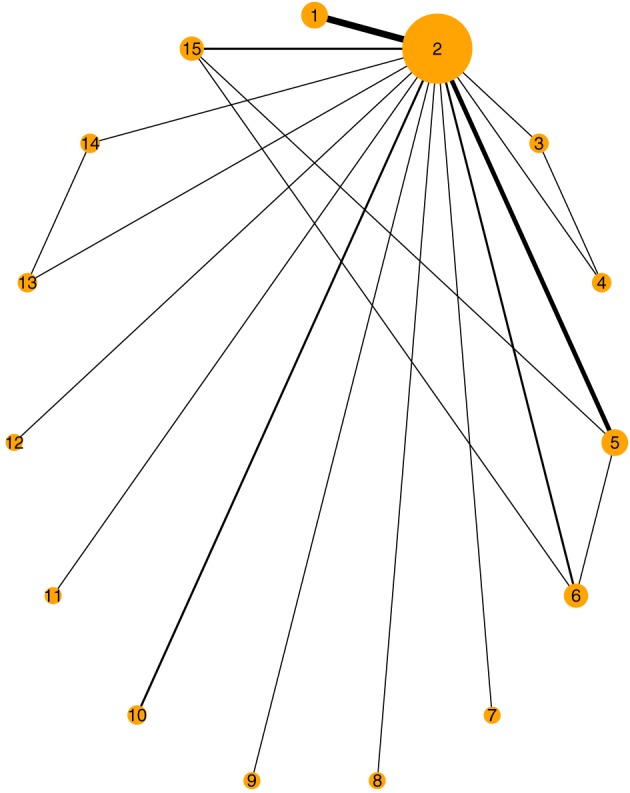
Network of trials of Stroke data [Colour figure can be viewed at http://wileyonlinelibrary.com]

Data have been collected and analyzed by Batson et al,^39^ and the raw data are given in the supporting information of their paper. Stroke data include 19 studies  that compare 15 different treatments to prevent stroke in patients with atrial fibrillation (AF). Treatments include fixed  low‐dose warfarin with or without aspirin, aspirin monotherapy, aspirin plus clopidogrel, indobufen, idraparinux, triflusal, and ximelagatran. The corresponding network plot is given in Figure 6. The primary outcome was the number of patients who had stroke events, a dichotomous  end point. The study‐level covariate of mean age is available. We fit a network meta‐regression model as described in Section 2.4 using both MCMC and INLA. Placebo was chosen to be the reference treatment. Note that since one study does not have the mean age information, the corresponding network meta‐regression model reduced in size by one (hence, 18 studies). In the network meta‐regression models, the interaction coefficient (β) is common for all treatment versus placebo. Table 4 displays the results of the fitted consistency and Jackson model with no covariate and with covariate information (mean age) using MCMC and INLA. Moreover, individual inconsistency random effects of the Jackson model without the covariate information are displayed in Table 5. Our results are in broad agreement with Figure 2 and Table 1 of Batson et al.^39^

**Table 4:** Quantiles of the marginal posterior distributions of basic parameters, heterogeneity, and inconsistency standard deviations by MCMC (top) and INLA (bottom) for Stroke application

	**No covariate**						**Covariate (age)**					
	**Consistency**			**Jackson**			**Consistency**			**Jackson**		
	Median	2.5*%*	97.5*%*	Median	2.5*%*	97.5*%*	Median	2.5*%*	97.5*%*	Median	2.5*%*	97.5*%*
**MCMC**												
*β*							0.02	−0.37	0.38	0.01	−0.37	0.37
*d*_1,2_	−1.23	−1.80	−0.72	−1.21	−3.10	0.78	−1.19	−2.41	−0.11	−1.20	−3.43	1.10
*d*_1,3_	−1.50	−2.50	−0.59	−1.48	−4.17	1.32	−1.44	−3.17	0.18	−1.46	−4.73	1.91
*d*_1,4_	−1.13	−2.10	−0.21	−1.10	−3.83	1.79	−1.07	−2.77	0.46	−1.06	−4.31	2.05
*d*_1,5_	−0.68	−1.50	0.18	−0.58	−2.86	2.10	−0.30	−2.13	1.26	−0.25	−3.02	2.77
*d*_1,6_	−0.11	−1.31	1.06	0.06	−2.05	3.15	0.03	−1.71	1.84	0.25	−2.34	3.60
*d*_1,7_	−0.61	−1.85	0.60	−0.64	−3.50	2.40	−0.55	−2.44	1.13	−0.58	−3.75	2.61
*d*_1,8_	−0.47	−1.45	0.49	−0.40	−3.18	2.50	−0.41	−2.12	1.16	−0.41	−3.65	2.83
**INLA**												
*β*							0.01	−0.35	0.36	0.01	−0.36	0.37
*d*_1,2_	−1.21	−1.79	−0.70	−1.22	−2.84	0.41	−1.19	−2.37	−0.17	−1.20	−3.34	0.90
*d*_1,3_	−1.48	−2.50	−0.55	−1.49	−3.86	0.89	−1.46	−3.13	0.04	−1.47	−4.52	1.55
*d*_1,4_	−1.10	−2.12	−0.17	−1.11	−3.48	1.26	−1.08	−2.75	0.41	−1.09	−4.14	1.92
*d*_1,5_	−0.67	−1.52	0.24	−0.59	−2.53	1.59	−0.31	−1.97	1.30	−0.28	−2.96	2.49
*d*_1,6_	−0.10	−1.29	1.14	0.03	−1.99	2.50	0.06	−1.54	1.81	0.21	−2.35	3.29
*d*_1,7_	−0.63	−1.85	0.58	−0.62	−3.07	1.85	−0.60	−2.38	1.07	−0.60	−3.71	2.49
*d*_1,8_	−0.42	−1.47	0.55	−0.43	−2.81	1.96	−0.40	−2.08	1.12	−0.41	−3.47	2.62
**MCMC**												
*d*_1,9_	−1.60	−2.74	−0.49	−1.55	−4.12	1.38	−1.55	−3.35	0.15	−1.55	−4.95	1.92
*d*_1,10_	−1.32	−2.16	−0.53	−1.28	−4.04	1.51	−1.27	−2.85	0.08	−1.29	−4.61	1.74
*d*_1,11_	−1.25	−2.21	−0.34	−1.22	−4.11	1.92	−1.20	−2.87	0.38	−1.21	−4.57	1.96
*d*_1,12_	−1.28	−2.27	−0.40	−1.26	−3.90	1.77	−1.22	−2.93	0.39	−1.24	−4.55	1.95
*d*_1,13_	−0.88	−1.86	0.05	−0.85	−3.44	2.04	−0.82	−2.55	0.77	−0.83	−4.03	2.40
*d*_1,14_	−1.24	−2.21	−0.36	−1.21	−3.84	1.69	−1.18	−2.92	0.38	−1.20	−4.37	1.95
*d*_1,15_	0.15	−0.78	1.06	0.18	−2.10	2.78	0.26	−1.40	1.73	0.31	−2.40	3.27
*τ*	0.23	0.01	0.84	0.27	0.01	0.96	0.37	0.02	1.30	0.38	0.02	1.31
*κ*				0.58	0.02	1.86				0.61	0.02	1.89
**INLA**												
*d*_1,9_	−1.57	−2.76	−0.44	−1.57	−4.01	0.86	−1.55	−3.32	0.07	−1.56	−4.66	1.51
*d*_1,10_	−1.28	−2.15	−0.49	−1.29	−3.61	1.04	−1.26	−2.73	0.04	−1.27	−4.22	1.65
*d*_1,11_	−1.23	−2.24	−0.30	−1.23	−3.60	1.14	−1.20	−2.87	0.29	−1.21	−4.26	1.80
*d*_1,12_	−1.26	−2.28	−0.33	−1.26	−3.64	1.11	−1.24	−2.90	0.26	−1.25	−4.30	1.77
*d*_1,13_	−0.85	−1.86	0.07	−0.86	−3.22	1.51	−0.83	−2.49	0.66	−0.84	−3.88	2.17
*d*_1,14_	−1.21	−2.22	−0.29	−1.21	−3.58	1.15	−1.19	−2.85	0.30	−1.20	−4.24	1.82
*d*_1,15_	0.16	−0.79	1.10	0.19	−1.82	2.32	0.26	−1.23	1.69	0.29	−2.30	2.98
*τ*	0.26	0.02	0.87	0.27	0.02	0.92	0.35	0.03	1.21	0.38	0.03	1.29
*κ*				0.55	0.03	1.61				0.65	0.03	1.85

Note: The first line shows the estimate for the interaction coefficient (*β*). INLA, integrated nested Laplace approximations; MCMC, Markov chain Monte Carlo.

**Table 5:** Estimated inconsistency parameters obtained from the fitted Jackson model for the Stroke dataset

		**MCMC**		**INLA**	
**Design**	**Parameter**	Mean	Stdev	Mean	Stdev
1	$\omega_{1,2}^{1}$	−0.05	0.86	−0.01	0.70
2	$\omega_{2,3}^{2}$	0.02	0.87	−0.00	0.70
	$\omega_{2,4}^{2}$	0.00	0.87	−0.00	0.70
3	$\omega_{2,5}^{3}$	−0.06	0.67	−0.03	0.57
4	$\omega_{2,6}^{3}$	−0.38	0.82	−0.00	0.70
	$\omega_{2,15}^{3}$	−0.14	0.69	−0.11	0.58
	$\omega_{2,5}^{4}$	−0.17	0.69	−0.15	0.56
5	$\omega_{2,7}^{5}$	0.01	0.85	−0.00	0.70
6	$\omega_{2,6}^{6}$	0.42	0.83	0.34	0.68
7	$\omega_{2,8}^{7}$	−0.01	0.86	−0.00	0.70
8	$\omega_{2,9}^{8}$	−0.04	0.87	−0.00	0.70
9	$\omega_{2,15}^{9}$	0.03	0.67	0.01	0.55
10	$\omega_{2,10}^{10}$	−0.01	0.84	−0.00	0.70
11	$\omega_{2,11}^{11}$	0.00	0.90	−0.00	0.70
12	$\omega_{2,12}^{12}$	−0.01	0.86	−0.00	0.70
13	$\omega_{2,13}^{13}$	−0.01	0.89	−0.00	0.70
	$\omega_{2,14}^{13}$	−0.01	0.87	−0.00	0.70

Abbreviations: INLA, integrated nested Laplace approximations; MCMC, Markov chain Monte Carlo.

From the Jackson model using INLA, the posterior median of the heterogeneity standard deviation is 0.27 with 95% CI [0.02, 0.92] suggesting moderate heterogeneity in the network. The posterior median of the inconsistency standard deviation is 0.55 with 95% CI [0.03, 1.61], suggesting there may be notable inconsistency with high uncertainty in the network. The DIC values of the consistency model  and the Jackson model without covariates are 283.05  and 283.72, respectively, which shows that adding inconsistency parameters does not result any improvement in DIC. From the results of individual inconsistency random effects, only $\omega_{26}^{3}$ and $\omega_{26}^{6}$ are relatively large but with very wide standard deviations. On the other hand, the addition of a covariate to the consistency model and the Jackson model, actually, increase the estimates of both heterogeneity and inconsistency standard deviations (*τ* and *κ*). The DIC values of the consistency model and the Jackson model with covariates are 271.31  and 272.05, respectively. To compare the models with covariate and without covariate information, we calculate the DIC values of the consistency model and the Jackson model without covariate when we drop the study, which does not have mean‐age information, and the results are 269.78  and 270.58, respectively. This suggests that adding covariates does not offer any notable improvement in the DIC values. Furthermore, from the results of the Jackson model with covariate, the posterior median estimate of *β* is 0.01 with 95% CI  [−0.36, 0.37]. Therefore, we can conclude that the inclusion of mean‐age covariate to the model fails to explain the source of heterogeneity and/or the inconsistency in the network.

Both methods MCMC and INLA gave similar results. Approximately, the MCMC run took 27.1  seconds, while INLA took only 5.4  seconds for the Jackson model with covariate.

## 5 DISCUSSION

We have presented an approximate Bayesian inference technique, INLA, to fit various contrast‐based NMA models with arm‐based likelihood including the Jackson model as well as their network meta‐regression extensions. Furthermore, to make it more accessible for researchers, we provide an R package, nmaINLA, which  automates INLA implementation of NMA models. There are good reasons to prefer INLA to MCMC. Firstly, INLA has better time performance. Secondly, there is no need to check any MCMC convergence diagnostics. Actually, this is very crucial for a large network, since the number of parameters to check diagnostics is increasing dramatically.

There is an ongoing debate about merits of the contrast‐based (CB) models and the arm‐based (AB) models. Relative treatment effect are assumed to be exchangeable across trials in the CB approach, whereas AB approach assumes that absolute treatment effects are exchangeable.^8^ The supporters of CB approach claim that “arm‐based pooling effectively breaks randomization, and in fact runs against the entire way in which randomized controlled trials are designed, analysed, and used .”^8^ AB modelers respond that “although AB models require different assumptions than CB models, it is not obvious that they are less reasonable, and the payoffs they can provide (significantly increased modeling flexibility, as well as greater ease of interpretation, prior specification, and model fitting) can be substantial.”^9^ For our concern, AB models are also in the family of  LGMs. Therefore, it is certainly possible to use INLA to fit AB models, although our package nmaINLA does not support AB models, yet. Alternative to the Jackson model, the node‐splitting method^10^ is another method to detect network inconsistencies. Although we have not discussed this method and not implemented it in nmaINLA; INLA of course could be used. The explanations and the necessary R‐code  are presented in Sauter and Held.^25^

One may find it restrictive to assume that heterogeneity and inconsistency random effects are normally distributed, hence explore different distributions for this assumption, for instance, *t* distribution.^42^ Although this  modeling approach is not in the scope of latent Gaussian models, INLA still can be used as an inference tool for such models.^43^

Unfortunately, there is no analytical expression for the approximation error obtained by INLA. A simple way to investigate its accuracy is comparison with long MCMC runs. The accuracy of INLA for fitting GLMMs has been investigated in rich simulation studies by Fong et al^31^ and Grilli et al.^44^ They reported INLA works very well in most cases, but in some extreme cases, of binary GLMMs with few or zero events per variable, INLA exhibits some inaccuracy. Moreover, for the special situation when a covariate (almost) perfectly predicts the response (quasicomplete separation) in binary response GLMMs, Sauter and Held^45^ have shown that the approximation error by INLA is substantial. Ferkingstad and Rue^46^ introduced a copula‐based correction, which significantly increase the accuracy of INLA for such extreme cases of GLMMs, and it is already implemented in R‐INLA. As a matter of fact, in the case of such “sparse data” situations^47^ of binary GLMM, it is known that both maximum likelihood methods and Bayesian inference with vague priors may result serious bias away from the null.^48^ Hence, different penalization techniques of maximum likelihood estimates (MLE) or using weakly informative priors for Bayesian inference have been advocated to avoid such biases.^49^ Such problems may occur in the NMA context as well, especially when the model is a binary GLMM. Therefore, network meta‐analyzer should be cautious not to obtain biased results regardless of his/her inference tool (MLE, MCMC, or INLA).

Using vague priors for hyperparameters of NMA models may make it extremely hard to identify these parameters. This can be overcome by using more informative priors. Using predictive distributions as priors for hyperparameters to fit the Jackson model is proposed by Law et al.^17^ On the other hand, Simpson et al^50^ has been introduced a principled and broad framework to construct prior distribution for a large class of hierarchical models. The priors that they develop, PC priors, are implemented in R‐INLA; hence, they can be used in a NMA context, especially for constructing priors for hyperparameters. Moreover, checking sensitivity of heterogeneity and inconsistency parameters to the chosen prior distributions may be particularly useful for NMA models. Although we did not discuss any sensitivity analysis, it can be easily conducted due to the computational speed of INLA.^51^ We note that the standard ranking of treatments as discussed in Lu and Ades^2^ is not possible using INLA. Although point estimates of ranking of treatments are provided, it is not possible to estimate the associated errors using INLA. This is because INLA is computing marginal posteriors but not joint posteriors. On the other hand, the standard ranking may be misleading since it does not take other evidences into account.^52^

## Supporting information



Listing 1: BUGS/JAGS code of the consistency model for dataset with dichotomous endpoints (Section 2.2 in the main text).
Listing 2: BUGS/JAGS code of the Jackson model for dataset with dichotomous endpoints (Section 2.3 in the main text).
Listing 3: BUGS/JAGS code of the network meta‐regression model for dataset with dichotomous endpoints (Section 2.4 in the main text).

R‐code for reproducing results of A design by‐treatment interaction model for network meta‐analysis with integrated nested Laplace approximations

Raw data used in A design by‐treatment interaction model for network meta‐analysis with integrated nested Laplace approximations

Figure 1: Network of trials of Smoking cessation.
Figure 2: Plot for the marginal posterior density of *d*_1,2_ and *τ* ^2^.

## References

[1] Lumley T . Network meta‐analysis for indirect treatment comparisons. Stat Med. 2002;21:2313‐2324. 12210616 10.1002/sim.1201

[2] Lu G , Ades AE . Assessing evidence inconsistency in mixed treatment comparisons. J Am Stat Assoc. 2006;101:447‐459.

[3] Higgins JPT , Thompson SG , Spiegelhalter DJ . A re‐evaluation of random‐effects meta‐analysis. J R Stat Soc Ser A Stat Soc. 2009;172:137‐159. 10.1111/j.1467-985X.2008.00552.x PMC2667312 19381330

[4] Salanti G . Indirect and mixed‐treatment comparison, network, or multiple‐treatments meta‐analysis: many names, many benefits, many concerns for the next generation evidence synthesis tool. Res Syn Meth. 2012;3:80‐97. 10.1002/jrsm.1037 26062083

[5] Higgins JPT , Welton NJ . Network meta‐analysis: a norm for comparative effectiveness?. Lancet. 2015;386:628‐630. 26334141 10.1016/S0140-6736(15)61478-7

[6] Hawkins N , Scott DA , Woods B . 'Arm‐based' parameterization for network meta‐analysis. Res Syn Meth. 2016;7:306‐313. 10.1002/jrsm.1187 PMC5063191 26610409

[7] Piepho HP . Network‐meta analysis made easy: detection of inconsistency using factorial analysis‐of‐variance models. BMC Med Res Methodol. 2014;14:1‐9. 24885590 10.1186/1471-2288-14-61 PMC4049370

[8] Dias S , Ades AE . Absolute or relative effects? Arm‐based synthesis of trial data. Res Syn Meth. 2016;7:23‐28. 10.1002/jrsm.1184 PMC5102631 26461457

[9] Hong H , Chu H , Zhang J , Carlin BP . Rejoinder to the discussion of “a Bayesian missing data framework for generalized multiple outcome mixed treatment comparisons” by S. Dias and A.E. Ades. Res Syn Meth. 2016;7:29‐33. 10.1002/jrsm.1186 PMC4779393 26461816

[10] Dias S , Welton NJ , Caldwell DM , Ades AE . Checking consistency in mixed treatment comparison meta‐analysis. Stat Med. 2010;29:932‐944. 20213715 10.1002/sim.3767

[11] Higgins JPT , Jackson D , Barrett JK , Lu G , Ades AE , White IR . Consistency and inconsistency in network meta‐analysis: concepts and models for multi‐arm studies. Res Syn Meth. 2012;3:98‐110. 10.1002/jrsm.1044 PMC4433772 26062084

[12] Jackson D , Barrett JK , Rice S , White IR , Higgins JPT . A design‐by‐treatment interaction model for network meta‐analysis with random inconsistency effects. Stat Med. 2014;33:3639‐3654. 24777711 10.1002/sim.6188 PMC4285290

[13] Dias S , Sutton AJ , Welton NJ , Ades AE . Evidence synthesis for decision making 3: Heterogeneity‐subgroups, meta‐regression, bias, and bias‐adjustment. MDM Policy Pract. 2013;33:618‐640. 10.1177/0272989X13485157 PMC3704206 23804507

[14] Jackson D , Law M , Barrett JK , et al. Extending Dersimonian and Laird's methodology to perform network meta‐analyses with random inconsistency effects. Stat Med. 2016;35:819‐839. 26423209 10.1002/sim.6752 PMC4973704

[15] Jackson D , Bujkiewicz S , Law M , Riley RD , White IR . A matrix‐based method of moments for fitting multivariate network meta‐analysis models with multiple outcomes and random inconsistency effects. Biometrics. 2017 https://doi.org/10.1111/biom.12762 10.1111/biom.12762 PMC6038911 28806485

[16] DerSimonian R , Laird N . Meta‐analysis in clinical trials. Control Clin Trials. 1986;7:177‐188. 3802833 10.1016/0197-2456(86)90046-2

[17] Law M , Jackson D , Turner R , Rhodes K , Viechtbauer W . Two new methods to fit models for network meta‐analysis with random inconsistency effects. BMC Med Res Methodol. 2016;16:87. 27465416 10.1186/s12874-016-0184-5 PMC4964019

[18] Jackson D , Veroniki A , Law M , Tricco AC , Baker R . Paule‐mandel estimators for network meta‐analysis with random inconsistency effects. Res Syn Meth. 2017;8:416‐434. 10.1002/jrsm.1244 PMC5720360 28585257

[19] Lunn DJ , Thomas A , Best N , Spiegelhalter D . WinBUGS—a Bayesian modelling framework: concepts, structure, and extensibility. Stat Comput. 2000;10:325‐337.

[20] Lunn D , Spiegelhalter D , Thomas A , Best N . The BUGS project: evolution, critique and future directions. Stat Med. 2009;28:3049‐3067. 19630097 10.1002/sim.3680

[21] Plummer M . JAGS: a program for analysis of Bayesian graphical models using Gibbs sampling. In: Proceedings of the 3rd International Workshop on Distributed Statistical Computing; 2003; Vienna, Austria. 1‐11.

[22] Stan Development Team . Stan modeling language user's guide and reference manual, version 2.12.0; 2016.

[23] Rue H , Martino S , Chopin N . Approximate Bayesian inference for latent Gaussian models by using integrated nested Laplace approximations. J R Stat Soc Series B Stat Methodol. 2009;71:319‐392.

[24] Paul M , Riebler A , Bachmann LM , Rue H , Held L . Bayesian bivariate meta‐analysis of diagnostic test studies using integrated nested Laplace approximations. Stat Med. 2010;29:1325‐1339. 20101670 10.1002/sim.3858

[25] Sauter R , Held L . Network meta‐analysis with integrated nested Laplace approximations. Biom J. 2015;57:1038‐1050. 26360927 10.1002/bimj.201400163

[26] Dias S , Welton NJ , Sutton AJ , Ades AE . NICE DSU Technical Support Document 2: A generalised linear modelling framework for pairwise and network meta‐analysis of randomised controlled trials. last updated September 2016; 2011. 27466657

[27] Higgins JPT , Whitehead A . Borrowing strength from external trials in a meta‐analysis. Stat Med. 1996;15:2733‐2749. 8981683 10.1002/(SICI)1097-0258(19961230)15:24<2733::AID-SIM562>3.0.CO;2-0

[28] Jackson D , Boddington P , White IR . The design‐by‐treatment interaction model: a unifying framework for modelling loop inconsistency in network meta‐analysis. Res Syn Meth. 2016;7:329‐332. 10.1002/jrsm.1188 PMC4946625 26588593

[29] Thompson SG , Higgins JPT . How should meta‐regression analyses be undertaken and interpreted?. Stat Med. 2002;21:1559‐1573. 12111920 10.1002/sim.1187

[30] Cooper NJ , Sutton AJ , Morris D , Ades AE , Welton NJ . Addressing between‐study heterogeneity and inconsistency in mixed treatment comparisons: application to stroke prevention treatments in individuals with non‐rheumatic atrial fibrillation. Stat Med. 2009;28:1861‐1881. 19399825 10.1002/sim.3594

[31] Fong Y , Rue H , Wakefield J . Bayesian inference for generalized linear mixed models. Biostatistics. 2010;11:397‐412. 19966070 10.1093/biostatistics/kxp053 PMC2883299

[32] Rue H , Riebler A , Sørbye SH , Illian JB , Simpson DP , Lindgren FK . Bayesian computing with INLA: a review. Annu Rev Stat Appl. 2017:395‐421.

[33] Tierney L , Kadane JB . Accurate approximations for posterior moments and marginal densities. J Am Stat Assoc. 1986;81:82‐86.

[34] Blangiardo M , Cameletti M . Bayesian computing Spatial and spatio‐temporal Bayesian models with R‐INLA. New York: John Wiley & Sons, Ltd; 2015:75‐126.

[35] Rue H , Riebler A , Sørbye SH , Illian JB , Simpson DP , Lindgren FK . Bayesian computing with inla: new features. Comput Stat Data Anal. 2013;67:68‐83.

[36] Spiegelhalter DJ , Best NG , Carlin BP , Van Der Linde A . Bayesian measures of model complexity and fit. J R Stat Soc Series B Stat Methodol. 2002;64:583‐639.

[37] Watanabe S . Asymptotic equivalence of bayes cross validation and widely applicable information criterion in singular learning theory. J Mach Learn Res. December 2010;11:3571‐3594.

[38] Su Y , Yajima M . R2jags: Using R to run 'JAGS'. R package version 0.5‐7; 2015.

[39] Batson S , Sutton A , Abrams K . Exploratory network meta regression analysis of stroke prevention in atrial fibrillation fails to identify any interactions with treatment effect. PLoS One. 2016;11(8):1‐12. e0161864. 10.1371/journal.pone.0161864 PMC4999289 27560191

[40] Gelman A , Rubin DB . Inference from iterative simulation using multiple sequences. Stat Sci. 1992;7:457‐472.

[41] Senn S , Gavini F , Magrez D , Scheen A . Issues in performing a network meta‐analysis. Stat Methods Med Res. 2013;22:169‐189. 22218368 10.1177/0962280211432220

[42] Lee KJ , Thompson SG . Flexible parametric models for random‐effects distributions. Stat Med. 2008;27:418‐434. 17477434 10.1002/sim.2897

[43] Martins TG , Rue H . Extending integrated nested Laplace approximation to a class of near‐Gaussian latent models. Scand Stat Theory Appl. 2014;41:893‐912.

[44] Grilli L , Metelli S , Rampichini C . Bayesian estimation with integrated nested Laplace approximation for binary logit mixed models. J Stat Comput Simul. 2015;85:2718‐2726.

[45] Sauter R , Held L . Quasi‐complete separation in random effects of binary response mixed models. J Stat Comput Simul. 2016;86:2781‐2796.

[46] Ferkingstad E , Rue H . Improving the inla approach for approximate bayesian inference for latent gaussian models. Electron J Stat. 2015;9:2706‐2731.

[47] Greenland S , Mansournia MA , Altman DG . Sparse data bias: a problem hiding in plain sight. BMJ. 2016;352:1‐6. 10.1136/bmj.i1981 27121591

[48] Gelman A , Jakulin A , Pittau MG , Su Y . A weakly informative default prior distribution for logistic and other regression models. Ann Appl Stat. 200812;2:1360‐1383.

[49] Greenland S , Mansournia MA . Penalization, bias reduction, and default priors in logistic and related categorical and survival regressions. Stat Med. 2015;34:3133‐3143. 26011599 10.1002/sim.6537

[50] Simpson D , Rue H , Riebler A , Martins TG , Sørbye SH . Penalising model component complexity: a principled, practical approach to constructing priors. Stat Sci. 2017;32:1‐28.

[51] Roos M , Martins TG , Held L , Rue H . Sensitivity analysis for Bayesian hierarchical models. Bayesian Anal. 201506;10:321‐349.

[52] Puhan MA , Schünemann HJ , Murad MH , et al. A grade working group approach for rating the quality of treatment effect estimates from network meta‐analysis. BMJ. 2014;349:1‐10. 10.1136/bmj.g5630 25252733

