# Supplementary material for: A design‐by‐treatment interaction model for network meta‐analysis and meta‐regression with integrated nested Laplace approximations
Source: Res Synth Methods. 2018 Jan 16;9(2):179–94. doi: 10.1002/jrsm.1285 (PMC6001639; doi:10.1002/jrsm.1285)
Supplement: Supplementary file 1 — Listing 1: BUGS/JAGS code of the consistency model for dataset with dichotomous endpoints (Section 2.2 in the main text). Listing 2: BUGS/JAGS code of the Jackson model for dataset with dichotomous endpoints (Section 2.3 in the main text). Listing 3: BUGS/JAGS code of the network meta‐regression model for dataset with dichotomous endpoints (Section 2.4 in the main text). [file JRSM-9-179-s001.pdf]

BUGS/JAGS code of the paper “A design-by-treatment interaction model for network meta-analysis with integrated nested Laplace approximations”

```
model{
  # LOOP THROUGH STUDIES
  for(i in 1:ns){
    # BINOMIAL LIKELIHOOD WITH LOGIT LINK
    # VAGUE PRIORS FOR TRIAL BASELINES
    base[i] ~ dnorm(meanf, precf)
    # LOOP THROUGH ARMS
    for (k in 1:na[i])  {
      r[i,k] ~ dbin(p[i,k],n[i,k])
      logit(p[i,k]) <- base[i] + eta[i,k]
    }
    # RANDOM EFFECTS DISTRIBUTION
    w[i,1] <- 0
    eta[i,1] <- 0
    for (k in 2:na[i]) {  # LOOP THROUGH ARMS
      eta[i,k] ~ dnorm(m.cond[i,k], prectau.cond[i,k])
      # MEANS WITH MULTI-ARM TRIAL CORRECTION
      m.cond[i,k] <-  delta[t[i,k]] - delta[t[i,1]] + sw[i,k]
      # BETWEEN-STUDY PRECISION WITH MULTI-ARM TRIAL CORRECTION
      prectau.cond[i,k] <- prectau * 2 * (k-1)/k
      w[i,k] <- (eta[i,k] - delta[t[i,k]] + delta[t[i,1]])
      sw[i,k] <- sum(w[i,1:(k-1)]) / (k-1)
    }
  }
    # TREATMENT EFFECT IS ZERO FOR REFERENCE TREATMENT
  delta[1] <- 0
  # VAGUE PRIORS
  for (k in 2:nt) { delta[k] ~ dnorm(meanf,precf) }
  stdtau  ~ dunif(0,ul)
  prectau <- pow(stdtau,-2)
  vartau <- 1 / prectau
}
```

Listing 1: BUGS/JAGS code of the consistency model for dataset with dichotomous endpoints (Section 2.2 in the main text).

```
model{
  # LOOP THROUGH STUDIES
  for(i in 1:ns){
    # BINOMIAL LIKELIHOOD WITH LOGIT LINK
    # VAGUE PRIORS FOR TRIAL BASELINES
    base[i] ~ dnorm(meanf, precf)
    # LOOP THROUGH ARMS
    for (k in 1:na[i])  {
      r[i,k] ~ dbin(p[i,k],n[i,k])
      logit(p[i,k]) <- base[i] + eta[i,k] + om[des[i], k]
    }
    # RANDOM EFFECTS DISTRIBUTION
    w[i,1] <- 0
    eta[i,1] <- 0
    for (k in 2:na[i]) {  # LOOP THROUGH ARMS
      eta[i,k] ~ dnorm(m.cond[i,k], prectau.cond[i,k])
      # MEANS WITH MULTI-ARM TRIAL CORRECTION
      m.cond[i,k] <-  delta[t[i,k]] - delta[t[i,1]] + sw[i,k]
      # BETWEEN-STUDY PRECISION WITH MULTI-ARM TRIAL CORRECTION
      prectau.cond[i,k] <- prectau * 2 * (k-1)/k
      w[i,k] <- (eta[i,k] - delta[t[i,k]] + delta[t[i,1]])
      sw[i,k] <- sum(w[i,1:(k-1)]) / (k-1)
    }
  }
  # INCONSISTENCY PARAMETERS
  for (i in 1:ndes)  {          # LOOP THROUGH DESIGNS
    om[i,1] <- 0
    for(k in 2:nades[i])  {  # LOOP THROUGH ARM OF DESIGN i
      om[i,k] ~ dnorm(mom.cond[i,k],precom.cond[i,k])
      # MEAN OF INCONSISTENCY DISTRIBUTION WITH MULTI-ARM TRIAL CORRECTION
      mom.cond[i,k] <- sum(om[i,1:(k-1)])/(k-1)
      # PRECISION OF INCONSISTENCY DISTRIBUTION WITH MULTI-ARM TRIAL CORRECTION
      precom.cond[i,k] <- preckappa * 2 * (k-1)/k
    }
  }

  # TREATMENT EFFECT IS ZERO FOR REFERENCE TREATMENT
  delta[1] <- 0
  # VAGUE PRIORS
  for (k in 2:nt) { delta[k] ~ dnorm(meanf,precf) }
  stdtau  ~  dunif(0,ul)
  prectau <- pow(stdtau,-2)
  vartau <- 1 / prectau
  stdkappa  ~  dunif(0,ul)
  preckappa <- pow(stdkappa,-2)
  varkappa <- 1 / preckappa
}
```

Listing 2: BUGS/JAGS code of the Jackson model for dataset with dichotomous endpoints (Section 2.3 in the main text).

```
model{
  # LOOP THROUGH STUDIES
  for(i in 1:ns){
  # BINOMIAL LIKELIHOOD WITH LOGIT LINK
  # VAGUE PRIORS FOR TRIAL BASELINES
  base[i] ~ dnorm(meanf, precf)
  # LOOP THROUGH ARMS
  for (k in 1:na[i])  {
    r[i,k] ~ dbin(p[i,k],n[i,k])
    logit(p[i,k]) <- base[i] + eta[i,k] + om[des[i], k] +
                     cov[i] * (beta[t[i,k]] - beta[t[i,1]])
  }
  # RANDOM EFFECTS DISTRIBUTION
  w[i,1] <- 0
  eta[i,1] <- 0
    for (k in 2:na[i]) {  # LOOP THROUGH ARMS
      eta[i,k] ~ dnorm(m.cond[i,k], prectau.cond[i,k])
      # MEANS WITH MULTI-ARM TRIAL CORRECTION
      m.cond[i,k] <-  delta[t[i,k]] - delta[t[i,1]] + sw[i,k]
      # BETWEEN-STUDY PRECISION WITH MULTI-ARM TRIAL CORRECTION
      prectau.cond[i,k] <- prectau * 2 * (k-1)/k
      w[i,k] <- (eta[i,k] - delta[t[i,k]] + delta[t[i,1]])
      sw[i,k] <- sum(w[i,1:(k-1)]) / (k-1)
    }
  }
  # INCONSISTENCY PARAMETERS
  for (i in 1:ndes)  {         # LOOP THROUGH DESIGNS
  om[i,1] <- 0
    for(k in 2:nades[i])  {  # LOOP THROUGH ARM OF DESIGN i
      om[i,k] ~ dnorm(mom.cond[i,k],precom.cond[i,k])
      # MEAN OF INCONSISTENCY DISTRIBUTION WITH MULTI-ARM TRIAL CORRECTION
      mom.cond[i,k] <- sum(om[i,1:(k-1)])/(k-1)
      # PRECISION OF INCONSISTENCY DISTRIBUTION WITH MULTI-ARM TRIAL CORRECTION
      precom.cond[i,k] <- preckappa * 2 * (k-1)/k
    }
  }
  # TREATMENT EFFECT IS ZERO FOR REFERENCE TREATMENT
  # COVARIATE IS ZERO FOR REFERENCE TREATMENT
  delta[1] <- 0
  beta[1] <- 0
  # VAGUE PRIORS
  for (k in 2:nt) {
    delta[k] ~ dnorm(meanf,precf)
    # common covariate effect
    beta[k] <- B
  }
  B ~ dnorm(meanf,precf)
  stdtau  ~  dunif(0,ul)
  prectau <- pow(stdtau,-2)
  vartau <- 1 / prectau
  stdkappa  ~  dunif(0,ul)
  preckappa <- pow(stdkappa,-2)
  varkappa <- 1 / preckappa
}
```

Listing 3: BUGS/JAGS code of the network meta-regression model for dataset with dichotomous endpoints (Section 2.4 in the main text).
