# Supplementary material for: A design‐by‐treatment interaction model for network meta‐analysis and meta‐regression with integrated nested Laplace approximations
Source: Res Synth Methods. 2018 Jan 16;9(2):179–94. doi: 10.1002/jrsm.1285 (PMC6001639; doi:10.1002/jrsm.1285)
Supplement: Supplementary file 2 — R‐code for reproducing results of A design by‐treatment interaction model for network meta‐analysis with integrated nested Laplace approximations [file JRSM-9-179-s002.pdf]

R-code for reproducing results of A design by-treatment interaction model for network meta-analysis with integrated nested Laplace approximations

0. Library and global parameter settings

purpose: load library and set up MCMC, INLA and prior distribution parameters

```
## 1. library
# Statistical analysis
# Install 'nmaINLA' package if it is not installed
# install.packages("nmaINLA")
library(nmaINLA)       # workhorse package
library(INLA)          # needed for 'nmaINLA'
library(R2jags)        # MCMC implementations
library(gemtc)         # used for Lu Ades and Jackson comparison
# Tables and figures
library(xtable)        # reproducible and nice tables
library(pcnetmeta)     # For network plots
library(ggplot2)       # nice figures
library(gridExtra)     # to plot ggplots in a same page
library(igraph)        # for illustrative network plots
#-----------------------------------------------------------------
set.seed(1234)


##=================================================================
## 2. MCMC parameters
##=================================================================
# MCMC settings
#burnin <- 100000
burnin <- 30000
burnin.at <- 50000
thin   <- 5
nchain <- 3
# Number of iterations for fixed effect and consitency models
n.iter.cons <- 20000 + burnin
n.iter.cons.at <- 50000 + burnin.at
# Number of iterations for Jackson models
n.iter.jack <- 50000 + burnin
n.iter.jack.at <- 100000 + burnin.at

##=================================================================
## 3. Fixed effect priors
##=================================================================
# Set the prior
fixed.par <- c(0, 1000)      # normal prior for fixed effects
```

```
##=================================================================
## 4. Random effect priors
##=================================================================
tau.par   <- c(0, 5)         # prior for tau in Section 4.1 and 4.2
kappa.par <- c(0, 5)         # prior for kappa in Section 4.1 and 4.2
# Set the prior for Stroke application
tau.par.atr   <- c(0, 2)        # prior for tau in Section 4.3
kappa.par.atr <- c(0, 2)        # prior for kappa in Section 4.3


##=================================================================
## 5. Function for saving the legend of ggplot
##=================================================================

get_legend<-function(myggplot){
  tmp <- ggplot_gtable(ggplot_build(myggplot))
  leg <- which(sapply(tmp$grobs, function(x) x$name) == "guide-box")
  legend <- tmp$grobs[[leg]]
  return(legend)
}
```

I. Code for Section 4.1 NMA for Diabetes application

purpose: reproducing results in Section 4.1

```
## 1. Load settings and data
#purpose:
#load R-packages and relevant R-functions and MCMC/INLA parameters.
#------------------------------------------------------------------------------------

## Diabetes application (Diabetesdat dataset)
data("Diabetesdat", package = "nmaINLA")
## data preparation
DiabetesdatINLA <- create_INLA_dat(dat = Diabetesdat,
                                   armVars = c('treatment' = 't', 'mean' = 'y',
                                               'std.err' = 'se'),
                                   design = "des", nArmsVar = 'na')

##=================================================================
## 2. Estimation with JAGS (MCMC)
##=================================================================
#purpose: estimate the NMA models with JAGS

#remarks:
#JAGS (together with R2jags) is used for MCMC sampling.
#The JAGS/BUGS-code is borrowed AND adaped from the JAGS-code
#given in Jackson et al (2014)
# JAGS codes are in the 'Jags.model' file.
# They are written in a '.txt' file (model.file), and given
```

```r
# to the 'R2jags::jags' function.
#----------------------------------------------------------------

# MCMC fixed effect
DiabetesdatfeJAGS <- list("ns" = nrow(Diabetesdat),
                          "nt" = max(Diabetesdat$t1, Diabetesdat$t2,
                                     Diabetesdat$t3, na.rm = TRUE),
                          "y" = cbind(Diabetesdat$y1, Diabetesdat$y2,
                                      Diabetesdat$y3),
                          "se" = cbind(Diabetesdat$se1, Diabetesdat$se2,
                                       Diabetesdat$se3),
                          "t" = cbind(Diabetesdat$t1, Diabetesdat$t2,
                                      Diabetesdat$t3),
                          "na" = Diabetesdat$na,
                          "precf" = 1 / fixed.par[2])

# By using R2jags
fit.Diabetes.FE.JAGS <- jags(data = DiabetesdatfeJAGS,
                             n.iter = n.iter.cons,
                             n.burnin = burnin,
                             n.thin = thin,
                             n.chains = nchain,
                             parameters.to.save = c("d"),
                             model.file = "Jags_models/JAGS.FE.NMA.cont.txt")

# random effects (consistency)
DiabetesdatreJAGS <- list("ns" = nrow(Diabetesdat),
                          "nt" = max(Diabetesdat$t1, Diabetesdat$t2, Diabetesdat$t3, na.rm = TRUE),
                          "y" = cbind(Diabetesdat$y1, Diabetesdat$y2, Diabetesdat$y3),
                          "se" = cbind(Diabetesdat$se1, Diabetesdat$se2, Diabetesdat$se3),
                          "t" = cbind(Diabetesdat$t1, Diabetesdat$t2, Diabetesdat$t3),
                          "na" = Diabetesdat$na,
                          "precf" = 1 / fixed.par[2],
                          "ul" = tau.par[2])

fit.Diabetes.RE.JAGS <- jags(data = DiabetesdatreJAGS,
                             n.iter = n.iter.cons,
                             n.burnin = burnin,
                             n.thin = thin,
                             n.chains = nchain,
                             parameters.to.save = c("d", "sd"),
                             model.file = "Jags_models/JAGS.RE.NMA.cont.txt")
# jackson model
DiabetesdatdesJAGS <- list("ns" = nrow(Diabetesdat),
                           "nt" = max(Diabetesdat$t1, Diabetesdat$t2, Diabetesdat$t3, na.rm = TRUE),
                           "y" = cbind(Diabetesdat$y1, Diabetesdat$y2, Diabetesdat$y3),
                           "se" = cbind(Diabetesdat$se1, Diabetesdat$se2, Diabetesdat$se3),
                           "t" = cbind(Diabetesdat$t1, Diabetesdat$t2, Diabetesdat$t3),
                           "na" = Diabetesdat$na,
                           "precf" = 1 / fixed.par[2],
                           "tau.par" = tau.par[2],
                           "kappa.par" = kappa.par[2],
                           "des" = Diabetesdat$des,
```

```
                           "nades" = c(2, 3, rep(2, 14)),
                           "ndes" = max(Diabetesdat$des))

# By using R2jags
sys.time.mcmc.Diabetes <-
  system.time(fit.Diabetes.DES.JAGS <- jags(data = DiabetesdatdesJAGS,
                                            n.iter = n.iter.jack,
                                            n.burnin = burnin,
                                            n.thin = thin,
                                            n.chains = nchain,
                                            parameters.to.save = c("d", "tau.sd", "kappa.sd"),
                                            model.file = "Jags_models/JAGS.DES.NMA.cont.txt"))

sys.time.mcmc.Diabetes.elapsed <- round(as.numeric(sys.time.mcmc.Diabetes[3]), 2)
##================================================================
## 3. Estimation with INLA
##================================================================
#purpose: estimate the NMA models with nmaINLA.
# type 'vignette('nmaINLA') for how to use nmaINLA package
#-----------------------------------------------------------------
# Fixed effect model
fit.Diabetes.FE.INLA <- nma_inla(DiabetesdatINLA, type = 'FE', likelihood = "normal",
                                 fixed.par = fixed.par)
# Consistency model
fit.Diabetes.RE.INLA <- nma_inla(DiabetesdatINLA, type = 'consistency',
                                 likelihood = "normal", fixed.par = fixed.par,
                                 tau.prior = "uniform", tau.par = tau.par)

# Jackson model
fit.Diabetes.DES.INLA <- nma_inla(DiabetesdatINLA, type = 'jackson',
                                  likelihood = 'normal', fixed.par = c(0, 1000),
                                  tau.prior = "uniform", tau.par = c(0, 5),
                                  kappa.prior = "uniform", kappa.par = c(0, 5))
print(fit.Diabetes.DES.INLA,2)

# Jackson model
sys.time.inla.Diabetes <-
  system.time(fit.Diabetes.DES.INLA <- nma_inla(DiabetesdatINLA, type = 'jackson',
                                                likelihood = "normal", fixed.par = fixed.par,
                                                tau.prior = "uniform", tau.par = tau.par,
                                                kappa.prior = "uniform", kappa.par = kappa.par))

sys.time.inla.Diabetes2 <-
  system.time(fit.Diabetes.DES.INLA2 <- nma_inla(DiabetesdatINLA, type = 'jackson',
                                                 likelihood = "normal", fixed.par = fixed.par,
                                                 tau.prior = "uniform", tau.par = tau.par,
                                                 kappa.prior = "uniform", kappa.par = kappa.par,
                                                 improve.hyperpar = FALSE))
sys.time.inla.Diabetes.elapsed <- round(as.numeric(sys.time.inla.Diabetes[3]), 2)
```

```
##==============================================================
## 4. Producing tables and plots
##==============================================================
#purpose:
# 1) create Figure 3 in the main text
# 2) create Table 1 in the main text
#--------------------------------------------------------------
JAGS.fe <- fit.Diabetes.FE.JAGS$BUGSoutput$summary[-c(1, 11), c(3, 5, 7)]
JAGS.cons <- fit.Diabetes.RE.JAGS$BUGSoutput$summary[-c(1, 11, 12), c(3, 5, 7)]
JAGS.des <- fit.Diabetes.DES.JAGS$BUGSoutput$summary[-c(1, 11, 12, 13), c(3, 5, 7)]

INLA.fe <- rbind(fit.Diabetes.FE.INLA$d_params[, c(3, 4, 5)])
INLA.cons <- rbind(fit.Diabetes.RE.INLA$d_params[, c(3, 4, 5)])
INLA.des <- rbind(fit.Diabetes.DES.INLA$d_params[, c(3, 4, 5)])

###
# PLOTTING
ests1  <- as.vector(rbind(JAGS.fe[,2], INLA.fe[, 2]))
lower1 <- as.vector(rbind(JAGS.fe[,1], INLA.fe[, 1]))
upper1 <- as.vector(rbind(JAGS.fe[,3], INLA.fe[, 3]))

d1 <- data.frame(x = ordered(1:18),
                 y = ests1,
                 ylo = lower1,
                 yhi = upper1,
                 c = rep(c("green", "red"), times = 9))

coef.plot1 <- ggplot(data = d1, aes(x = x, y = y, ymin = ylo, ymax = yhi, colour = c)) +
  geom_pointrange(show.legend = TRUE, fatten = 2) +
  scale_colour_manual(values = c("blue","red"), labels = c("MCMC 95%-CI", "INLA 95%-CI")) +
  geom_hline(aes(yintercept = 0), lty = 2) +
  ylab("") +
  xlab("") +
  scale_x_discrete(labels = c(expression(d[12]), expression(d[13]),
                              expression(d[14]), expression(d[15]),
                              expression(d[16]), expression(d[17]),
                              expression(d[18]), expression(d[19]),
                              expression(d[110])),
                   breaks = seq(1, 18, 2),
                   drop = TRUE)  +
  scale_y_continuous(limits = c(-1.75, 0.48)) +
  theme(axis.text.x = element_text(size = 10, colour = "black"),
        plot.title = element_text(lineheight=.8, face="bold", hjust = 0.5)) +
  theme(legend.title = element_blank(), legend.position = "bottom", legend.direction = "horizontal",
        legend.text = element_text(size = 10, face = "bold"), legend.key.height = unit(1.2, "cm")) +
  ggtitle("Fixed effect model")

# consistency model
ests2  <- as.vector(rbind(JAGS.cons[,2], INLA.cons[, 2]))
lower2 <- as.vector(rbind(JAGS.cons[,1], INLA.cons[, 1]))
upper2 <- as.vector(rbind(JAGS.cons[,3], INLA.cons[, 3]))

d2 <- data.frame(x = ordered(1:18),
```

```
                 y = ests2,
                 ylo = lower2,
                 yhi = upper2,
                 c = rep(c("green", "red"), times = 9))

coef.plot2 <- ggplot(data = d2, aes(x = x, y = y, ymin = ylo, ymax = yhi, colour = c)) +
  geom_pointrange(show.legend = FALSE, fatten = 2) +
  scale_colour_manual(values = c("blue","red"), labels = c("MCMC 95%-CI", "INLA 95%-CI")) +
  geom_hline(aes(yintercept = 0), lty = 2) +
  ylab("") +
  xlab("") +
  scale_x_discrete(labels = c(expression(d[12]), expression(d[13]),
                              expression(d[14]), expression(d[15]),
                              expression(d[16]), expression(d[17]),
                              expression(d[18]), expression(d[19]),
                              expression(d[110])),
                   breaks = seq(1, 18, 2),
                   drop = TRUE)  +
  scale_y_continuous(limits = c(-1.75, 0.48)) +
  theme(axis.text.x = element_text(size = 10, colour = "black"),
        plot.title = element_text(lineheight=.8, face="bold", hjust = 0.5)) +
  ggtitle("Consistency model")

# jackson model
ests3  <- as.vector(rbind(JAGS.des[,2], INLA.des[, 2]))
lower3 <- as.vector(rbind(JAGS.des[,1], INLA.des[, 1]))
upper3 <- as.vector(rbind(JAGS.des[,3], INLA.des[, 3]))

d3 <- data.frame(x = ordered(1:18),
                 y = ests3,
                 ylo = lower3,
                 yhi = upper3,
                 c = rep(c("green", "red"), times = 9))

coef.plot3 <- ggplot(data = d3, aes(x = x, y = y, ymin = ylo, ymax = yhi, colour = c)) +
  geom_pointrange(show.legend = FALSE, fatten = 2) +
  scale_colour_manual(values = c("blue","red"), labels = c("MCMC 95%-CI", "INLA 95%-CI")) +
  geom_hline(aes(yintercept = 0), lty = 2) +
  ylab("") +
  xlab("") +
  scale_x_discrete(labels = c(expression(d[12]), expression(d[13]),
                              expression(d[14]), expression(d[15]),
                              expression(d[16]), expression(d[17]),
                              expression(d[18]), expression(d[19]),
                              expression(d[110])),
                   breaks = seq(1, 18, 2),
                   drop = TRUE)  +
  scale_y_continuous(limits = c(-1.75, 0.48)) +
  theme(axis.text.x = element_text(size = 10, colour = "black"),
        plot.title = element_text(lineheight=.8, face="bold", hjust = 0.5)) +
  ggtitle("Jackson model")

# Save the legend
```

```
legend_Diabetes <- get_legend(coef.plot1)
# Remove the legend from the plot_d12
coef.plot1 <- coef.plot1 + theme(legend.position="none")
# Save plot as .eps
print(grid.arrange(coef.plot1, coef.plot2, coef.plot3, legend_Diabetes,
                   ncol = 1, nrow = 4, heights = c(1, 1, 1, 0.5)))

####
# TABLE 1
####
### Hyperparameters
tau_cons_mcmc  <- fit.Diabetes.RE.JAGS$BUGSoutput$summary[12, 5]
tauL_cons_mcmc <- fit.Diabetes.RE.JAGS$BUGSoutput$summary[12, 3]
tauU_cons_mcmc <- fit.Diabetes.RE.JAGS$BUGSoutput$summary[12, 7]
tau_jack_mcmc  <- fit.Diabetes.DES.JAGS$BUGSoutput$summary[13, 5]
tauL_jack_mcmc <- fit.Diabetes.DES.JAGS$BUGSoutput$summary[13, 3]
tauU_jack_mcmc <- fit.Diabetes.DES.JAGS$BUGSoutput$summary[13, 7]
kappa_jack_mcmc  <- fit.Diabetes.DES.JAGS$BUGSoutput$summary[12, 5]
kappaL_jack_mcmc <- fit.Diabetes.DES.JAGS$BUGSoutput$summary[12, 3]
kappaU_jack_mcmc <- fit.Diabetes.DES.JAGS$BUGSoutput$summary[12, 7]
# INLA
tau_cons_inla  <- fit.Diabetes.RE.INLA$hyperpar[, 4]
tauL_cons_inla <-  fit.Diabetes.RE.INLA$hyperpar[, 3]
tauU_cons_inla <-  fit.Diabetes.RE.INLA$hyperpar[, 5]
tau_jack_inla  <-  fit.Diabetes.DES.INLA$hyperpar[1, 4]
tauL_jack_inla <-  fit.Diabetes.DES.INLA$hyperpar[1, 3]
tauU_jack_inla <-  fit.Diabetes.DES.INLA$hyperpar[1, 5]
kappa_jack_inla  <-  fit.Diabetes.DES.INLA$hyperpar[2, 4]
kappaL_jack_inla <-  fit.Diabetes.DES.INLA$hyperpar[2, 3]
kappaU_jack_inla <-  fit.Diabetes.DES.INLA$hyperpar[2, 5]
# creating table
cons_mcmc <- c(NA, tau_cons_mcmc, tauL_cons_mcmc, tauU_cons_mcmc, NA, NA, NA, NA)
cons_inla <- c(NA, tau_cons_inla, tauL_cons_inla, tauU_cons_inla, NA, NA, NA, NA)
jack_mcmc <- c(NA, tau_jack_mcmc, tauL_jack_mcmc, tauU_jack_mcmc, NA, kappa_jack_mcmc, kappaL_jack_mcmc
jack_inla <- c(NA, tau_jack_inla, tauL_jack_inla, tauU_jack_inla, NA, kappa_jack_inla, kappaL_jack_inla
results <- cbind(cons_mcmc, cons_inla, jack_mcmc, jack_inla)
rownames(results) <- c(
  "\\textbf{Heterogeneity ($\\tau$)}",
  "Posterior median",
  "Lower b.(95\\%-CI)",
  "Upper b.(95\\%-CI)",
  "\\textbf{Inconsistency ($\\kappa$)}",
  "Posterior median",
  "Lower b.(95\\%-CI)",
  "Upper b.(95\\%-CI)"
)
#prepare orientation /indent of column text:
alig <- c("lcccc")
#print xtable produces Latex-Table:
print(
  xtable(
    as.data.frame(results),
    digits=3,
```

```
    align=paste(alig),
    label="t01:Diabetes",
    caption="Estimates of heterogeneity and inconsistency standard
    deviation of consistency and Jackson model for the Diabetes application.") ,
  caption.placement="top",
  table.placement="htb",
  booktabs = TRUE,
  sanitize.text.function = function(x){x},  #replace special characters
  include.rownames=TRUE,
  include.colnames=FALSE,
  add.to.row=list(pos=list(0,0),  #add multicolumns
                  command=c(" &\\multicolumn{2}{c}{\\textbf{Consistency}} &
\\multicolumn{2}{c}{\\textbf{Jackson}}  \\\\
                             \\cmidrule(rl){2-3} \\cmidrule(rl){4-5}",
                             " & MCMC & INLA & MCMC & INLA \\\\"
                  ))
)

###################
# Largest absolute difference
###################
round(max(abs(JAGS.fe[1, 2] - INLA.fe[1, 2]), abs(JAGS.fe[2, 2] - INLA.fe[2, 2]),
          abs(JAGS.fe[3, 2] -  INLA.fe[3, 2]), abs(JAGS.fe[4, 2] - INLA.fe[4, 2]),
          abs(JAGS.fe[5, 2] - INLA.fe[5, 2]), abs(JAGS.fe[6, 2] - INLA.fe[6, 2]),
          abs(JAGS.fe[7, 2] - INLA.fe[7, 2]), abs(JAGS.fe[8, 2] - INLA.fe[8, 2]),
          abs(JAGS.fe[9, 2] - INLA.fe[9, 2]),

          abs(JAGS.cons[1, 2] - INLA.cons[1, 2]),
          abs(JAGS.cons[2, 2] - INLA.cons[2, 2]), abs(JAGS.cons[3, 2] - INLA.cons[3, 2]),
          abs(JAGS.cons[4, 2] - INLA.cons[4, 2]), abs(JAGS.cons[5, 2] - INLA.cons[5, 2]),
          abs(JAGS.cons[6, 2] - INLA.cons[6, 2]), abs(JAGS.cons[7, 2] - INLA.cons[7, 2]),
          abs(JAGS.cons[8, 2] - INLA.cons[8, 2]), abs(JAGS.cons[9, 2] - INLA.cons[9, 2]),

          abs(JAGS.des[1, 2] - INLA.des[1, 2]), abs(JAGS.des[2, 2] - INLA.des[2, 2]),
          abs(JAGS.des[3, 2] - INLA.des[3, 2]), abs(JAGS.des[4, 2] - INLA.des[4, 2]),
          abs(JAGS.des[5, 2] - INLA.des[5, 2]), abs(JAGS.des[6, 2] - INLA.des[6, 2]),
          abs(JAGS.des[7, 2] - INLA.des[7, 2]), abs(JAGS.des[8, 2] - INLA.des[8, 2]),
          abs(JAGS.des[9, 2] - INLA.des[9, 2]) ), 4)
```

II. Code for Section 4.2 NMA for Smoking application

purpose: reproducing results in Section 4.2

```
## 1.  Load settings and data
##==================================================================================
#purpose:
#load R-packages and relevant R-functions and MCMC/INLA parameters.
#----------------------------------------------------------------------------------
#load data:
data("Smokdat", package = "nmaINLA")
```

```
SmokdatINLA <- create_INLA_dat(dat = Smokdat,
                               armVars = c('treatment' = 't', 'responders' = 'r',
                                           'sampleSize' = 'n'),
                               design = "des", nArmsVar = 'na')


##=================================================================
## 2. Estimation with gemtc-JAGS (MCMC).
##=================================================================
#purpose: estimate the Jackson model with JAGS

#remarks:
#JAGS (together with R2jags) is used for MCMC sampling.
#The JAGS/BUGS-code is borrowed AND adaped from the JAGS-code
#given in Jackson et al (2014)
# JAGS codes are in the 'Jags.model' file.
# They are written in a '.txt' file (model.file), and given
# to the 'R2jags::jags' function.
#-----------------------------------------------------------------

jags.Smoking.inc <- list("nt" = 4,
                         "ns" = 24,
                         "ndes" = 8,
                         "nades" = c(3, 3, 2, 2, 2, 2, 2, 2),
                         "r" = cbind(Smokdat$r1, Smokdat$r2, Smokdat$r3),
                         "n" = cbind(Smokdat$n1, Smokdat$n2, Smokdat$n3),
                         "t" = cbind(Smokdat$t1, Smokdat$t2, Smokdat$t3),
                         "na" = Smokdat$na,
                         "des" = Smokdat$des,
                         "precf" = 1 / fixed.par[2],
                         "meanf" = fixed.par[1],
                         "ul" =  tau.par[2]
)

params.Smoking.inc <- c("delta[2:4]", "stdtau", "vartau", "stdkappa", "varkappa",
                        "om[1:8, 2]", "om[1:2, 3]", "base", "eta[1:24, 2]", "eta[1:2, 3]")

# Run JAGS model:
sys.time.mcmc.smok <-
  system.time(jags.smokeREinc.JAGS <- jags(data = jags.Smoking.inc,
                                           n.iter = n.iter.jack,
                                           n.burnin = burnin,
                                           n.thin = thin,
                                           n.chains = nchain,
                                           parameters.to.save = params.Smoking.inc,
                                           model.file =
                                             "Jags_models/JAGS.Design.unifprior.Model.txt"))
sys.time.mcmc.smok.elapsed <- round(as.numeric(sys.time.mcmc.smok[3]), 2)

# To look at the MC standard error, use runjags
#library(runjags)
#sys.time.runjags.smok <- system.time(jags.smokeREinc.runJAGS <- run.jags(data = jags.Smoking.inc,
#                                                                  sample = 80000 - burnin,
```

```
#                                                                burnin = burnin,
#                                                                thin = thin,
#                                                                n.chains = nchain,
#                                                                monitor = params.Smoking.inc,
#                                                                model = "Jags_models/JAGS.Design.unifpr
#sys.time.runjags.smok.elapsed <- round(as.numeric(sys.time.runjags.smok[3]), 2)

#summary_runjags <- summary(jags.smokeREinc.runJAGS)
#max(summary_runjags[, 7])
##=================================================================
## 3. Estimation with INLA.
##=================================================================
#purpose: estimate the Jackson model with nmaINLA.
# type 'vignette('nmaINLA') for how to use nmaINLA package
#-----------------------------------------------------------------

sys.time.inla.smok <-
  system.time(inla.smokeDesinc <- nma_inla(SmokdatINLA, likelihood = "binomial",
                                            fixed.par = fixed.par,
                                            tau.prior = "uniform", tau.par = tau.par,
                                            kappa.prior = "uniform", kappa.par = tau.par,
                                            type = "jackson", improve.hyperpar.dz = 0.5))
sys.time.inla.smok.elapsed <- round(as.numeric(sys.time.inla.smok[3]), 2)

# Without inla.hyperpar
sys.time.inla.smok2 <-
  system.time(inla.smokeDesinc2 <- nma_inla(SmokdatINLA, likelihood = "binomial",
                                             fixed.par = fixed.par,
                                             tau.prior = "uniform", tau.par = tau.par,
                                             kappa.prior = "uniform", kappa.par = tau.par,
                                             type = "jackson", improve.hyperpar.dz = FALSE))
sys.time.inla.smok.elapsed2 <- round(as.numeric(sys.time.inla.smok2[3]), 2)


# Inconsistency parameters
JAGS.Des.inc.params <- jags.smokeREinc.JAGS$BUGSoutput$summary[5:14, ][, c(1, 2)]
inla.inc.ref <- inla.smokeDesinc$summary.random$inc[c(1, 9, 2, 10, 3:8),][, c(2, 3)]

##=================================================================
## 4. Producing tables and plots.
##=================================================================
#purpose: create Figure 5 and table 2 in the paper.
#-----------------------------------------------------------------

mcmc.Smoking.inc    <- as.mcmc(jags.smokeREinc.JAGS)
# Plot of the marginal posterior distributions
# d12
# Suitable objects for ggplot2 plots!
d12.inla <- data.frame(inla.smarginal(marginal = inla.smokeDesinc$marginals.fixed$d12))
d12.mcmc <- data.frame(mcmc.Smoking.inc[[1]][, "delta[2]"])
# The first plot with legend
plot_d12 <- ggplot(data = d12.mcmc, aes(x = var1)) +
  geom_histogram(aes(y = ..density.., fill = "MCMC"), colour = "gray48", bins = 50) +
```

```r
  geom_path(data = d12.inla, aes(x = x, y = y, colour = "INLA")) +
  xlab(expression(paste(d[12]))) +
  ylab(" ") +
  scale_colour_manual(" ", values = c("INLA" = "black") ) +
  scale_fill_manual(" ", breaks = "MCMC", values = c("red"))
# d13
d13.inla <- data.frame(inla.smarginal(marginal = inla.smokeDesinc$marginals.fixed$d13))
d13.mcmc <- data.frame(mcmc.Smoking.inc[[1]][, "delta[3]"])
plot_d13 <- ggplot(data = d13.mcmc, aes(x = var1)) +
  geom_histogram(aes(y = ..density..), fill = "red", colour = "gray48", bins = 70) +
  geom_path(data = d13.inla, aes(x = x, y = y), colour = "black") +
  xlab(expression(paste(d[13]))) +
  ylab(" ")
# d14
d14.inla <- data.frame(inla.smarginal(marginal = inla.smokeDesinc$marginals.fixed$d14))
d14.mcmc <- data.frame(mcmc.Smoking.inc[[1]][, "delta[4]"])
plot_d14 <- ggplot(data = d14.mcmc, aes(x = var1)) +
  geom_histogram(aes(y = ..density..), fill = "red", colour = "gray48", bins = 50) +
  geom_path(data = d14.inla, aes(x = x, y = y), colour = "black") +
  xlab(expression(paste(d[14]))) +
  ylab(" ")
# Hyperparameters
# tau2 : heterogeneity variance not tau!
# tau2 : heterogeneity variance not tau!
prec.post.reinc <- inla.smokeDesinc$marginals.hyperpar$`Precision for het`
tau2.inla <- data.frame(inla.tmarginal(function(x) 1/x, prec.post.reinc, n = 20000))
# MCMC
tau2.mcmc <- data.frame(mcmc.Smoking.inc[[1]][, "vartau"])
# The combined plot
plot_tau2 <- ggplot(data = tau2.mcmc, aes(x = var1)) +
  geom_histogram(aes(y = ..density..), fill = "red", colour = "gray48", bins = 100) +
  geom_path(data = tau2.inla, aes(x = x, y = y), colour = "black") +
  coord_cartesian(xlim = c(0, 2.8)) +
  xlab(expression(paste(tau^2))) +
  ylab(" ")
# kappa2 : heterogeneity variance not kappa!
prec.post.reinc <- inla.smokeDesinc$marginals.hyperpar$`Precision for inc`
kappa2.inla <- data.frame(inla.tmarginal(function(x) 1/x, prec.post.reinc, n = 2000))
kappa2.mcmc <- data.frame(mcmc.Smoking.inc[[1]][, "varkappa"])
# The combined plot
plot_kappa2 <- ggplot(data = kappa2.mcmc, aes(x = var1)) +
  geom_histogram(aes(y = ..density..), fill = "red", colour = "gray48", bins = 500) +
  geom_path(data = kappa2.inla, aes(x = x, y = y), colour = "black") +
  coord_cartesian(xlim = c(0, 2.8), ylim = c(0, 6)) +
  xlab(expression(paste(kappa^2))) +
  ylab(" ")
# Save the legend
legend_smok <- get_legend(plot_d12)

# Remove the legend from the plot_d12
plot_d12 <- plot_d12 + theme(legend.position="none")

postscript(file="Figure_2.eps",horiz=FALSE,onefile=FALSE,width=8.5,height=11,paper='letter')
```

```
print(grid.arrange(plot_d12, plot_d13, plot_d14, legend_smok, plot_tau2,
                   plot_kappa2, ncol=2, nrow = 3))
dev.off()

####
# TABLE 2
####
Design <- c("1", NA, "2", NA, "3", "4", "5", "6", "7", "8")
Parameter <- c("$\\omega_{13}^1$", "$\\omega_{14}^1$", "$\\omega_{23}^2$",
               "$\\omega_{24}^2$", "$\\omega_{13}^3$"
               , "$\\omega_{12}^4$", "$\\omega_{14}^5$", "$\\omega_{23}^6$",
               "$\\omega_{24}^7$", "$\\omega_{34}^8$")
results <- data.frame(Design, Parameter, round(JAGS.Des.inc.params[, 1], digits = 2),
                      round(JAGS.Des.inc.params[, 2], digits = 2),
                      round(inla.inc.ref[, 1], digits = 2), round(inla.inc.ref[, 2],
                                                                  digits = 2))

# prepare orientation /indent of column text:
aligt2 <- c('l', 'c', 'c', rep('r', 4))
# print xtable produces Latex-Table:
myxtable <- xtable(results,
                   align = paste(aligt2),
                   label = "t3:incparams",
                   caption = "Estimated inconsistency parameters obtained from the
                   fitted Jackson model for the Smoking dataset.")
print(myxtable,
      caption.placement = "top",
      table.placement="htb",
      booktabs = TRUE,
      sanitize.text.function = function(x){x},  # replace special characters
      include.rownames = FALSE,
      include.colnames = FALSE,
      add.to.row = list(pos = list(0, 0),  # add multicolumns
                        command = c("\\textbf{Design} & \\textbf{Parameter}  &
\\multicolumn{2}{c}{\\textbf{MCMC}} & \\multicolumn{2}{c}{\\textbf{INLA}} \\\\
                                    \\cmidrule(rl){3-4} \\cmidrule(rl){5-6}",
                                    " &  & Mean & Stdev. & Mean & Stdev. \\\\"
                        )
      )
      )

#######
# Absolute difference
#######
round(max(abs(JAGS.Des.inc.params[1, 1] - inla.inc.ref[1, 1]),
          abs(JAGS.Des.inc.params[2, 1] - inla.inc.ref[2, 1]),
          abs(JAGS.Des.inc.params[3, 1] - inla.inc.ref[3, 1]),
          abs(JAGS.Des.inc.params[4, 1] - inla.inc.ref[4, 1]),
          abs(JAGS.Des.inc.params[5, 1] - inla.inc.ref[5, 1]),
          abs(JAGS.Des.inc.params[6, 1] - inla.inc.ref[6, 1]),
          abs(JAGS.Des.inc.params[7, 1] - inla.inc.ref[7, 1]),
          abs(JAGS.Des.inc.params[8, 1] - inla.inc.ref[8, 1]),
          abs(JAGS.Des.inc.params[9, 1] - inla.inc.ref[9, 1]),
```

```
        abs(JAGS.Des.inc.params[10, 1] - inla.inc.ref[10, 1])), digits = 3)
#
```

III. Code for Section 4.3 NMA for Stroke application

purpose: reproducing results in Section 4.3

```
##==========================================================================================
## 1.  Load settings and data, and data preparation
##==========================================================================================
#purpose:
#load R-packages and relevant R-functions and MCMC/INLA parameters.
#------------------------------------------------------------------------------------------
#load data:
data("Strokedat", package = "nmaINLA")
# data preparation for INLA
StrokedatINLA <- create_INLA_dat(dat = Strokedat,
                                 armVars = c('treatment' = 't', 'responders' = 'r' ,
                                             'sampleSize' = 'n'),
                                 design = 'des',
                                 nArmsVar = 'na')
## dataset for NMA regression
# Delete 13th study!, since there is no covariate information
Strokedat.mreg <- Strokedat[-c(13),]
# centering the covariate
Strokedat.mreg$age <- Strokedat.mreg$age - mean(Strokedat.mreg$age)

# data preparation for INLA
StrokedatINLA.mreg <- create_INLA_dat(dat = Strokedat.mreg,
                                      armVars = c('treatment' = 't', 'responders' = 'r' ,
                                                  'sampleSize' = 'n'),
                                      covariate = 'age',
                                      design = 'des',
                                      nArmsVar = 'na')


##=================================================================
## 2. Estimation with gemtc-JAGS (MCMC).
##=================================================================
#purpose: estimate the Consistency model, Jackson model and the
#Consistency model with covariate, Jackson model  with covariate via JAGS

#remarks:
#JAGS (together with R2jags) is used for MCMC sampling.
#The JAGS/BUGS-code is borrowed AND adaped from the JAGS-code
#given in Jackson et al (2014)
# JAGS codes are in the 'Jags.model' file.
# They are written in a '.txt' file (model.file), and given
# to the 'R2jags::jags' function.
#-----------------------------------------------------------------
```

```r
## Consistency
# MCMC

# MCMC
StrokedatJAGS <- list("nt" = max(c(Strokedat$t1, Strokedat$t2, Strokedat$t3, Strokedat$t4,
                                  Strokedat$t5), na.rm = TRUE),
                      "ns" = nrow(Strokedat),
                      "r" = cbind(Strokedat$r1, Strokedat$r2, Strokedat$r3, Strokedat$r4,
                                 Strokedat$r5),
                      "n" = cbind(Strokedat$n1, Strokedat$n2, Strokedat$n3, Strokedat$n4,
                                 Strokedat$n5),
                      "t" = cbind(Strokedat$t1, Strokedat$t2, Strokedat$t3, Strokedat$t4,
                                 Strokedat$t5),
                      "na" = Strokedat$na,
                      "meanf" = fixed.par[1],
                      "precf" = 1 / fixed.par[2],
                      "ul" = tau.par.atr[2])

# By using R2jags
fit.Stroke.CONS.JAGS <- jags(data = StrokedatJAGS,
                             n.iter = n.iter.cons.at,
                             n.burnin = burnin.at,
                             n.thin = thin,
                             n.chains = nchain,
                             parameters.to.save = c("delta", "stdtau"),
                             model.file = "Jags_models/JAGS.Cons.unifprior.Model.txt")


# MCMC
StrokedatJAGS <- list("nt" = max(c(Strokedat.mreg$t1, Strokedat.mreg$t2, Strokedat.mreg$t3,
                                  Strokedat.mreg$t4, Strokedat.mreg$t5), na.rm = TRUE),
                      "ns" = nrow(Strokedat.mreg),
                      "r" = cbind(Strokedat.mreg$r1, Strokedat.mreg$r2, Strokedat.mreg$r3,
                                 Strokedat.mreg$r4, Strokedat.mreg$r5),
                      "n" = cbind(Strokedat.mreg$n1, Strokedat.mreg$n2, Strokedat.mreg$n3,
                                 Strokedat.mreg$n4, Strokedat.mreg$n5),
                      "t" = cbind(Strokedat.mreg$t1, Strokedat.mreg$t2, Strokedat.mreg$t3,
                                 Strokedat.mreg$t4, Strokedat.mreg$t5),
                      "na" = Strokedat.mreg$na,
                      "cov" = Strokedat.mreg$age, # centered covariates
                      "meanf" = fixed.par[1],
                      "precf" = 1 / fixed.par[2],
                      "ul" = tau.par.atr[2]) # centered covariates

# By using R2jags
fit.Stroke.CONS.MREG.JAGS <- jags(data = StrokedatJAGS,
                                  n.iter = n.iter.cons,
                                  n.burnin = burnin,
                                  n.thin = thin,
                                  n.chains = nchain,
                                  parameters.to.save = c("delta", "stdtau", "B"),
                                  model.file = "Jags_models/JAGS.Cons.MREG.Model.txt")
```

```r
########################
### Jackson model
########################

## Without covariate

# results

# MCMC
StrokedatJAGS <- list("nt" = max(c(Strokedat$t1, Strokedat$t2, Strokedat$t3, Strokedat$t4,
                                  Strokedat$t5), na.rm = TRUE),
                      "ns" = nrow(Strokedat),
                      "r" = cbind(Strokedat$r1, Strokedat$r2, Strokedat$r3, Strokedat$r4,
                                 Strokedat$r5),
                      "n" = cbind(Strokedat$n1, Strokedat$n2, Strokedat$n3, Strokedat$n4,
                                 Strokedat$n5),
                      "t" = cbind(Strokedat$t1, Strokedat$t2, Strokedat$t3, Strokedat$t4,
                                 Strokedat$t5),
                      "na" = Strokedat$na,
                      "meanf" = fixed.par[1],
                      "precf" = 1 / fixed.par[2],
                      "ul" = tau.par.atr[2],
                      "ndes" = max(Strokedat$des),
                      "nades" =  c(2, 3, 4, rep(2, times = 9), 3),
                      "des" = Strokedat$des)

# By using R2jags
fit.Stroke.JACK.JAGS <- jags(data = StrokedatJAGS,
                             n.iter = n.iter.jack,
                             n.burnin = burnin,
                             n.thin = thin,
                             n.chains = nchain,
                             parameters.to.save = c("delta", "stdtau", "stdkappa"),
                             model.file = "Jags_models/JAGS.Design.unifprior.Model.txt")

# MCMC
StrokedatJAGS <- list("nt" = max(c(Strokedat.mreg$t1, Strokedat.mreg$t2, Strokedat.mreg$t3,
                                  Strokedat.mreg$t4, Strokedat.mreg$t5), na.rm = TRUE),
                      "ns" = nrow(Strokedat.mreg),
                      "r" = cbind(Strokedat.mreg$r1, Strokedat.mreg$r2, Strokedat.mreg$r3,
                                 Strokedat.mreg$r4, Strokedat.mreg$r5),
                      "n" = cbind(Strokedat.mreg$n1, Strokedat.mreg$n2, Strokedat.mreg$n3,
                                 Strokedat.mreg$n4, Strokedat.mreg$n5),
                      "t" = cbind(Strokedat.mreg$t1, Strokedat.mreg$t2, Strokedat.mreg$t3,
                                 Strokedat.mreg$t4, Strokedat.mreg$t5),
                      "na" = Strokedat.mreg$na,
                      "meanf" = fixed.par[1],
                      "precf" = 1 / fixed.par[2],
                      "cov" = Strokedat.mreg$age, # centered covariates
                      "ul" = tau.par.atr[2],
                      "ndes" = max(Strokedat.mreg$des),
                      "nades" =  c(2, 3, 4, rep(2, times = 9), 3),
                      "des" = Strokedat.mreg$des)
```

```r
# By using R2jags
sys.time.mcmc.atr <-
  system.time(fit.Stroke.JACK.MREG.JAGS <- jags(data = StrokedatJAGS,
                                                n.iter = n.iter.jack,
                                                n.burnin = burnin,
                                                n.thin = thin,
                                                n.chains = nchain,
                                                parameters.to.save = c("delta", "stdtau",
                                                                       "stdkappa", "B"),
                                                model.file =
                                                  "Jags_models/JAGS.Design.MREG.Model.txt"))
sys.time.mcmc.atr.elapsed <- round(as.numeric(sys.time.mcmc.atr[3]), 2)

##================================================================
## 3. Estimation with INLA.
##================================================================
#purpose: estimate the Jackson model with nmaINLA.
# type 'vignette('nmaINLA') for how to use nmaINLA package
#----------------------------------------------------------------

fit.Stroke.CONS.INLA <- nma_inla(StrokedatINLA, likelihood = "binomial", fixed.par = fixed.par,
                                 tau.prior = "uniform",
                                 tau.par = tau.par, type = 'consistency')
## With covariate

# INLA
fit.Stroke.CONS.MREG.INLA <- nma_inla(StrokedatINLA.mreg, likelihood = "binomial",
                                      fixed.par = fixed.par, tau.prior = "uniform",
                                      tau.par = tau.par.atr, type = 'consistency', mreg = TRUE)

# INLA
fit.Stroke.JACK.INLA <- nma_inla(StrokedatINLA, fixed.par = fixed.par, tau.prior = "uniform",
                                 tau.par = tau.par.atr, kappa.prior = "uniform",
                                 kappa.par = kappa.par.atr, type = 'jackson', likelihood = "binomial",
                                 improve.hyperpar.dz = 0.5)

## With covariate

# INLA
sys.time.inla.atr <-
  system.time(fit.Stroke.JACK.MREG.INLA <- nma_inla(StrokedatINLA.mreg,
                                                    fixed.par = fixed.par,
                                                    tau.prior = "uniform",
                                                    tau.par = tau.par.atr, kappa.prior = "uniform",
                                                    kappa.par = kappa.par.atr, type = 'jackson',
                                                    mreg = TRUE,
                                                    likelihood = "binomial",
                                                    improve.hyperpar.dz = 0.5))
sys.time.inla.atr.elapsed <- round(as.numeric(sys.time.inla.atr[3]), 2)

##================================================================
## 4. Producing tables and plots.
##================================================================
```

```r
#purpose: create Figure 5 and table 2 in the paper.
#----------------------------------------------------------------

JAGS.cons <- fit.Stroke.CONS.JAGS$BUGSoutput$summary[c(2:15, 17),][, c(3, 5, 7)]
INLA.cons <- rbind(fit.Stroke.CONS.INLA$d_params[, c(3, 4, 5)],
                   fit.Stroke.CONS.INLA$hyperpar[, c(3, 4, 5)])
# Covariate
# consistency model
JAGS.mreg <- fit.Stroke.CONS.MREG.JAGS$BUGSoutput$summary[c(1, 3:16, 18),][, c(3, 5, 7)]
INLA.mreg <- rbind(fit.Stroke.CONS.MREG.INLA$cov[, c(3, 4, 5)],
                   fit.Stroke.CONS.MREG.INLA$d_params[, c(3, 4, 5)],
                   fit.Stroke.CONS.MREG.INLA$hyperpar[, c(3, 4, 5)])
# Jackson model
JAGS.Des <- fit.Stroke.JACK.JAGS$BUGSoutput$summary[c(2:15, 18, 17),][, c(3, 5, 7)]
INLA.Des <- rbind(fit.Stroke.JACK.INLA$d_params[, c(3, 4, 5)],
                  fit.Stroke.JACK.INLA$hyperpar[1, c(3, 4, 5)],
                  fit.Stroke.JACK.INLA$hyperpar[2, c(3, 4, 5)])
# covariate
JAGS.Des.mreg <- fit.Stroke.JACK.MREG.JAGS$BUGSoutput$summary[c(1, 3:16, 19, 18),][, c(3, 5, 7)]
INLA.Des.mreg <- rbind(fit.Stroke.JACK.MREG.INLA$cov[, c(3, 4, 5)],
                       fit.Stroke.JACK.MREG.INLA$d_params[, c(3, 4, 5)],
                       fit.Stroke.JACK.MREG.INLA$hyperpar[1, c(3, 4, 5)],
                       fit.Stroke.JACK.MREG.INLA$hyperpar[2, c(3, 4, 5)])
# Results
median.cons  <- c(NA, NA, JAGS.cons[, 2], NA, NA, NA, INLA.cons[, 2], NA)
medianL.cons <- c(NA, NA, JAGS.cons[, 1], NA, NA, NA, INLA.cons[, 1], NA)
medianU.cons <- c(NA, NA, JAGS.cons[, 3], NA, NA, NA, INLA.cons[, 3], NA)
median.des   <- c(NA, NA, JAGS.Des[, 2], NA, NA, INLA.Des[, 2])
medianL.des  <- c(NA, NA, JAGS.Des[, 1], NA, NA, INLA.Des[, 1])
medianU.des  <- c(NA, NA, JAGS.Des[, 3], NA, NA, INLA.Des[, 3])
median.mreg   <- c(NA, JAGS.mreg[, 2], NA, NA, INLA.mreg[, 2], NA)
medianL.mreg  <- c(NA, JAGS.mreg[, 1], NA, NA, INLA.mreg[, 1], NA)
medianU.mreg  <- c(NA, JAGS.mreg[, 3], NA, NA, INLA.mreg[, 3], NA)
median.des.mreg   <- c(NA, JAGS.Des.mreg[, 2], NA, INLA.Des.mreg[, 2])
medianL.des.mreg  <- c(NA, JAGS.Des.mreg[, 1], NA, INLA.Des.mreg[, 1])
medianU.des.mreg  <- c(NA, JAGS.Des.mreg[, 3], NA, INLA.Des.mreg[, 3])

results <- cbind(median.cons, medianL.cons, medianU.cons,
                 median.des, medianL.des, medianU.des,
                 median.mreg, medianL.mreg, medianU.mreg,
                 median.des.mreg, medianL.des.mreg, medianU.des.mreg)

# Table 1
results1 <- results[c(1:9, 19:27),]
rownames(results1) <- c(
  "\\textbf{MCMC}",
  "$\\beta$",
  "$d_{1,2}$",
  "$d_{1,3}$",
  "$d_{1,4}$",
  "$d_{1,5}$",
  "$d_{1,6}$",
  "$d_{1,7}$",
```

```
  "$d_{1,8}$",
  "\\textbf{INLA}",
  "$\\beta$",
  "$d_{1,2}$",
  "$d_{1,3}$",
  "$d_{1,4}$",
  "$d_{1,5}$",
  "$d_{1,6}$",
  "$d_{1,7}$",
  "$d_{1,8}$")

# prepare orientation /indent of column text:
aligt2 <- c("lrrrrrrrrrrrr")
# print xtable produces Latex-Table:
myxtable1 <- xtable(as.data.frame(results1),
                    digits = 2,
                    align = paste(aligt2),
                    label = "t5:nmareg1",
                    caption = "Quantiles of the marginal posterior distributions
                    of basic parameters, heterogeneity and inconsistency standard
                    deviations by MCMC (top) and INLA (bottom) for AF application.
                    The first line shows the estimate for the interaction coefficient ($\\beta$).")
print(myxtable1,
      hline.after = getOption("xtable.hline.after", c(-1, 0, 9, nrow(myxtable1))),
      caption.placement = "top",
      table.placement="htb",
      booktabs = TRUE,
      sanitize.text.function = function(x){x},  # replace special characters
      floating.environment = "sidewaystable",
      include.rownames = TRUE,
      include.colnames = FALSE,
      add.to.row = list(pos = list(0, 0, 0),  # add multicolumns
                        command = c("&\\multicolumn{6}{c}{\\textbf{No covariate}}&
                              \\multicolumn{6}{c}{\\textbf{Covariate (age)}} \\\\
                              \\cmidrule(rl){2-7} \\cmidrule(rl){8-13}",
                               "& \\multicolumn{3}{c}{\\textbf{Consistency}} &
                              \\multicolumn{3}{c}{\\textbf{Jackson}}&
                              \\multicolumn{3}{c}{\\textbf{Consistency}} &
                              \\multicolumn{3}{c}{\\textbf{Jackson}} \\\\
                              \\cmidrule(rl){2-4} \\cmidrule(rl){5-7}
                              \\cmidrule(rl){8-10} \\cmidrule(rl){11-13}",
                              "& Median & $2.5 \\%$ & $97.5 \\%$ & Median &
                              $2.5 \\%$ & $97.5 \\%$ & Median & $2.5 \\%$ & $97.5
                              \\%$ & Median & $2.5 \\%$ & $97.5 \\%$ \\\\")
                        )
                        )

results2 <- rbind(NA, results[10:18,], NA, results[28:36,] )
rownames(results2) <- c(
  "\\textbf{MCMC}",
  "$d_{1,9}$",
  "$d_{1,10}$",
  "$d_{1,11}$",
```

```
  "$d_{1,12}$",
  "$d_{1,13}$",
  "$d_{1,14}$",
  "$d_{1,15}$",
  "$\\tau$",
  "$\\kappa$",
  "\\textbf{INLA}",
  "$d_{1,9}$",
  "$d_{1,10}$",
  "$d_{1,11}$",
  "$d_{1,12}$",
  "$d_{1,13}$",
  "$d_{1,14}$",
  "$d_{1,15}$",
  "$\\tau$",
  "$\\kappa$"
)
# prepare orientation /indent of column text:
aligt2 <- c("lrrrrrrrrrrrr")
# print xtable produces Latex-Table:
myxtable2 <- xtable(as.data.frame(results2),
                    digits = 2,
                    align = paste(aligt2),
                    label = "t5:nmareg2",
                    caption = "Continued.")
print(myxtable2,
      hline.after = getOption("xtable.hline.after", c(-1, 0, 10, nrow(myxtable2))),
      caption.placement = "top",
      table.placement="htb",
      booktabs = TRUE,
      sanitize.text.function = function(x){x},  # replace special characters
      floating.environment = "sidewaystable",
      include.rownames = TRUE,
      include.colnames = FALSE,
      add.to.row = list(pos = list(0, 0, 0),  # add multicolumns
                        command = c("&\\multicolumn{6}{c}{\\textbf{No covariate}}&
\\multicolumn{6}{c}{\\textbf{Covariate (age)}} \\\\
                                    \\cmidrule(rl){2-7} \\cmidrule(rl){8-13}",
                                    "& \\multicolumn{3}{c}{\\textbf{Consistency}} &
\\multicolumn{3}{c}{\\textbf{Jackson}}&
                                    \\multicolumn{3}{c}{\\textbf{Consistency}} &
\\multicolumn{3}{c}{\\textbf{Jackson}} \\\\
                                    \\cmidrule(rl){2-4} \\cmidrule(rl){5-7}
                                    \\cmidrule(rl){8-10} \\cmidrule(rl){11-13}",
                                    "& Median & $2.5 \\%$ & $97.5 \\%$ & Median & $2.5
                                    \\%$ & $97.5 \\%$ & Median & $2.5 \\%$ & $97.5 \\%$
                                    & Median & $2.5 \\%$ & $97.5 \\%$ \\\\")
      )
)
```
