# Supplementary material for: A design‐by‐treatment interaction model for network meta‐analysis and meta‐regression with integrated nested Laplace approximations
Source: Res Synth Methods. 2018 Jan 16;9(2):179–94. doi: 10.1002/jrsm.1285 (PMC6001639; doi:10.1002/jrsm.1285)
Supplement: Supplementary file 3 — Raw data used in A design by‐treatment interaction model for network meta‐analysis with integrated nested Laplace approximations [file JRSM-9-179-s003.pdf]

Raw data used in A design by-treatment interaction model for network meta-analysis with integrated nested Laplace approximations

Diabetes application in Section 4.1

Results from 26 trials examining the effectiveness of glucose-lowering agents in patients with type 2 diabetes. `y1, y2 and y3` are the sample means; `se1, se2 and se3` are the associated standar errors, `t1, t2 and t3` are the coding of treatments, in the first, second and third treatments arms, respectively. `na` is the number of arms, `des` is the design of each study.

y1	y2	y3	se1	se2	se3	t1	t2	t3	na	des
0.20	-1.70	NA	0.100	0.100	NA	1	2	NA	2	1
0.08	-0.74	NA	0.084	0.053	NA	1	2	NA	2	1
-1.30	-2.30	-2.5	0.340	0.320	0.16	1	5	2	3	2
0.14	-1.20	NA	0.101	0.101	NA	1	3	NA	2	3
0.20	-0.90	NA	0.080	0.081	NA	1	3	NA	2	3
0.10	-1.20	NA	0.076	0.101	NA	1	4	NA	2	4
-0.14	-0.91	NA	0.074	0.078	NA	1	3	NA	2	3
7.45	7.61	NA	0.060	0.060	NA	2	4	NA	2	5
6.70	6.80	NA	0.139	0.119	NA	3	4	NA	2	6
0.10	-1.20	NA	0.068	0.075	NA	1	3	NA	2	3
-0.08	-1.17	NA	0.160	0.160	NA	1	3	NA	2	3
-0.40	-1.90	NA	0.127	0.101	NA	1	3	NA	2	3
-0.95	-1.09	NA	0.153	0.163	NA	2	3	NA	2	7
-1.10	0.10	NA	0.122	0.076	NA	3	7	NA	2	8
-0.70	-0.30	NA	0.125	0.091	NA	5	7	NA	2	9
-0.30	-1.10	NA	0.130	0.060	NA	1	5	NA	2	10
0.27	-0.30	NA	0.091	0.091	NA	1	8	NA	2	11
0.07	-0.63	NA	0.090	0.090	NA	1	9	NA	2	12
0.13	0.50	NA	0.084	0.084	NA	2	7	NA	2	13
0.33	-0.41	NA	0.130	0.130	NA	1	6	NA	2	14
0.98	-0.43	NA	0.200	0.100	NA	1	6	NA	2	14
-1.10	-1.10	NA	0.152	0.178	NA	2	3	NA	2	15
0.56	-0.12	NA	0.200	0.200	NA	1	6	NA	2	14
-0.90	-1.30	NA	0.296	0.319	NA	1	2	NA	2	1
8.26	8.03	NA	0.246	0.244	NA	1	10	NA	2	16
0.19	-0.82	NA	0.110	0.081	NA	1	10	NA	2	16

Smoking cessation application in Section 4.2

The smoking cessation dataset describes a network comparing 4 treatments with 24 trials. There are 22 pairwise comparisons and 2 thee-arm trials. `r1, r2 and r3` are the number of observed events; `n1, n2 and n3` are the sample sizes, `t1, t2 and t3` are the coding of treatments, in the first, second and third treatments arms, respectively. `na` is the number of arms, `des` is the design of each study.

r1	r2	r3	n1	n2	n3	t1	t2	t3	na	des
9	23	10	140	140	138	1	3	4	3	1
11	12	29	78	85	170	2	3	4	3	2
75	363	NA	731	714	1	1	3	NA	2	3
2	9	NA	106	205	1	1	3	NA	2	3
58	237	NA	549	1561	1	1	3	NA	2	3
0	9	NA	33	48	1	1	3	NA	2	3
3	31	NA	100	98	1	1	3	NA	2	3
1	26	NA	31	95	1	1	3	NA	2	3
6	17	NA	39	77	1	1	3	NA	2	3
79	77	NA	702	694	1	1	2	NA	2	4
18	21	NA	671	535	1	1	2	NA	2	4
64	107	NA	642	761	1	1	3	NA	2	3
5	8	NA	62	90	1	1	3	NA	2	3
20	34	NA	234	237	1	1	3	NA	2	3
0	9	NA	20	20	1	1	4	NA	2	5
8	19	NA	116	146	1	1	2	NA	2	4
95	143	NA	1107	1031	1	1	3	NA	2	3
15	35	NA	187	504	1	1	3	NA	2	3
78	73	NA	584	675	1	1	3	NA	2	3
69	54	NA	1177	888	1	1	3	NA	2	3
20	16	NA	49	43	1	2	3	NA	2	6
7	32	NA	66	127	1	2	4	NA	2	7
12	20	NA	76	74	1	3	4	NA	2	8
9	3	NA	55	26	1	3	4	NA	2	8

Stroke prevention application in Section 4.3

Dataset for network meta-regression of stroke prevention in Atrial Fibrillation. A total of 19 studies, and primary endpoint is reported ischaemic stroke. It includes 15 comparators which include fixed low dose warfarin with or without aspirin, aspirin monotherapy, aspirin plus clopidogrel, indobufen, idraparinux, triflusal and ximelagatran. Study level covariate is the mean age (`age`). `r1, r2, r3 and r4` are the number of observed events; `n1, n2, n3 and n4` are the sample sizes, `t1, t2 , t3 and t4` are the coding of treatments, in the first, second, third and fourth treatments arms, respectively. `na` is the number of arms, `des` is the design of each study.

r1	n1	r2	n2	r3	n3	r4	n4	t1	t2	t3	t4	na	des	age
9	181	5	187	NA	1	NA	1	1	2	NA	NA	2	1	67.50
143	6022	111	6076	159	6015	NA	1	2	3	4	NA	3	2	71.66
19	265	4	260	NA	1	NA	1	1	2	NA	NA	2	1	67.00
3	170	5	169	5	167	8	171	2	5	6	15	4	3	73.25
2	131	4	141	NA	1	NA	1	2	5	NA	NA	2	4	70.50
10	454	18	462	NA	1	NA	1	2	7	NA	NA	2	5	72.50
0	153	5	152	NA	1	NA	1	2	6	NA	NA	2	6	74.50
42	3371	90	3335	NA	1	NA	1	2	8	NA	NA	2	7	70.00
20	2107	13	1922	NA	1	NA	1	2	9	NA	NA	2	8	70.00
13	208	2	212	NA	1	NA	1	1	2	NA	NA	2	1	68.50
39	214	16	225	NA	1	NA	1	1	2	NA	NA	2	1	70.50
17	211	6	210	NA	1	NA	1	1	2	NA	NA	2	1	65.50
26	555	37	545	NA	1	NA	1	2	5	NA	NA	2	4	NA
11	523	43	521	NA	1	NA	1	2	15	NA	NA	2	9	71.50

r1	n1	r2	n2	r3	n3	r4	n4	t1	t2	t3	t4	na	des	age
46	1703	32	1704	NA	1	NA	1	2	10	NA	NA	2	10	70.00
36	1962	45	1960	NA	1	NA	1	2	10	NA	NA	2	10	72.00
221	7090	218	7081	NA	1	NA	1	2	11	NA	NA	2	11	71.00
155	9081	149	9120	NA	1	NA	1	2	12	NA	NA	2	12	70.00
235	7036	333	7034	236	7035	NA	1	2	13	14	NA	3	13	71.66
