# Supplementary material for: A design‐by‐treatment interaction model for network meta‐analysis and meta‐regression with integrated nested Laplace approximations
Source: Res Synth Methods. 2018 Jan 16;9(2):179–94. doi: 10.1002/jrsm.1285 (PMC6001639; doi:10.1002/jrsm.1285)
Supplement: Supplementary file 4 — Figure 1: Network of trials of Smoking cessation. Figure 2: Plot for the marginal posterior density of d 1,2 and τ 2. [file JRSM-9-179-s004.pdf]

Supplementary material to "A design-by-treatment interaction model for network meta-analysis with integrated nested Laplace approximations"

Burak Kürsad Günhan,[a] [b] Tim Friede,[a] Leonhard Held[c]

This document contains additional information for the paper "A design-by-treatment interaction model for network meta-analysis with integrated nested Laplace approximations". INLA methodology is implemented as an R (R Core Team, 2016) package `INLA` (http://www.r-inla.org/), hereafter `R-INLA`. Our package `nmaINLA` is a purpose-built front end of the `R-INLA`. While `R-INLA` offers full Bayesian inference for the large set of latent Gaussian models using integrated nested Laplace approximations, `nmaINLA` extracts the features needed for many NMA models and presents them in an intuitive way. The purpose of this document is to demonstrate how to use `nmaINLA`.

1 Installation

Firstly, `R-INLA` package should be installed. Note that `R-INLA` is not on the Comprehensive R Archive Network (CRAN). We recommend the testing version of `R-INLA`. It can be downloaded from INLA website (http://www.r-inla.org/). Or it can be installed (and loaded) with following commands:

```
install.packages("INLA", repos = "https://www.math.ntnu.no/inla/R/testing")
library("INLA")
```

The stable version of `nmaINLA` is on CRAN (https://CRAN.R-project.org/package=nmaINLA). `nmaINLA` can be installed as follows:

```
install.packages("nmaINLA")
```

2 Fitting Consistency and Jackson models

Here, we use the Smoking application which is discussed Section 4.2 of the main text.

```
library("nmaINLA")
data("Smokdat", package = "nmaINLA")
head(Smokdat)

##    r1  r2 r3  n1   n2  n3 t1 t2 t3 na des
## 1   9  23 10 140  140 138  1  3  4  3   1
## 2  11  12 29  78   85 170  2  3  4  3   2
## 3  75 363 NA 731  714   1  1  3 NA  2   3
## 4   2   9 NA 106  205   1  1  3 NA  2   3
```

[a] *Department of Medical Statistics, University Medical Center Göttingen, Göttingen, Germany*
[b] *Correspondence to: Burak Kürsad Günhan; email:* `burak.gunhan@med.uni-goettingen.de`
[c] *Epidemiology, Biostatistics and Prevention Institute, University of Zurich, Zurich, Switzerland*

```
## 5 58 237 NA 549 1561    1   1   3  NA   2    3
## 6  0    9 NA  33   48    1   1   3  NA   2    3
```

Additional information can be obtained by typing `?Smokdat` (for any dataset and function in the package). As one can notice, the form of dataset is one-study-per-row format. This format is widely used and convenient for BUGS models. The only different covariate is `des` which is the vector of *designs*. That variable is only needed to fit Jackson model and should be added by "hand" to the dataset. Then, this dataset should be converted to one-arm-per-row format, and some indicator variables should be added as well. This can be done using `create_INLA_dat` function:

```
SmokdatINLA <- create_INLA_dat(dat = Smokdat,
                               armVars = c('treatment' = 't', 'responders' = 'r',
                                           'sampleSize' = 'n'),
                               nArmsVar = 'na',
                               design = 'des')
head(SmokdatINLA)

##   study treatment responders sampleSize na baseline mu d12 d13 d14  g het
## 1     1         1          9        140  3        1  1   0   0   0 NA  NA
## 2     1         3         23        140  3        1  1   0   1   0  1   1
## 3     1         4         10        138  3        1  1   0   0   1  2   1
## 4     2         2         11         78  3        2  2   0   0   0 NA  NA
## 5     2         3         12         85  3        2  2  -1   1   0  1   2
## 6     2         4         29        170  3        2  2  -1   0   1  2   2
##   inc
## 1  NA
## 2   1
## 3   1
## 4  NA
## 5   2
## 6   2
```

Figure 1 (a network plot) can be created using `plot_nma`:

```
plot_nma(s.id = study, t.id = treatment, data = SmokdatINLA)
```

`nma_inla` is the main fitting function of this package. It is actually a wrapper for `inla` function from `R-INLA`. Since Smoking dataset has binomial endpoints, the consistency model can be fitted by specifying `likelihood = "binomial"` as follows:

```
fit.consistency <- nma_inla(SmokdatINLA, likelihood = "binomial",
                            fixed.par = c(0, 1000), tau.prior = "uniform",
                            tau.par = c(0, 5), type = "consistency")
```

Note that only Normal prior is available for priors of fixed effects of the model (including baseline risks and basic parameters). A simple summary of the fitted model is given by `print` option:

```
print(fit.consistency)

## Network meta-analysis using INLA
## Relative treatment effects
##      mean    sd 0.025quant 0.5quant 0.975quant
## d12 0.494 0.401     -0.285    0.488      1.308
## d13 0.842 0.238      0.391    0.834      1.337
## d14 1.101 0.437      0.268    1.090      1.998
## Heterogeneity stdev
##       mean         sd 0.025quant   0.5quant 0.975quant
##      0.838      0.183      0.546      0.814      1.268
```

For post-processing, R-INLA functions can be used. A plot for the marginal posterior density of basic parameter ($d_{1,2}$) can be plotted using `inla.smarginal` `R-INLA` function (see Figure 2A).

```
d12.inla <- inla.smarginal(marginal = fit.consistency$marginals.fixed$d12)
plot(d12.inla, type = "l", xlab = expression(paste(d[12])), ylab = " ")
```

`R-INLA` internally uses precisions (on logarithmic scale) for the posterior marginals of hyperparameters (corresponds to τ for a Consistency model). To obtain variances instead of logarithm of precisions of heterogeneity, transformation of the hyperparameter is needed. Transformation and plotting can be done as follows (see Figure 2B):

```
log.prec.het <- fit.consistency$internal.marginals.hyperpar$`Log precision for het`
tau2.inla <- inla.tmarginal(function(x) 1/exp(x), log.prec.het, n = 20000)
plot(tau2.inla, type = "l", xlab = expression(paste(tau)), ylab = " ")
```

Finally, the Jackson model can be fitted by specifying `type = 'jackson'`:

```
fit.jackson <- nma_inla(SmokdatINLA, likelihood = "binomial",
                        fixed.par = c(0, 1000), tau.prior = "uniform",
                        tau.par = c(0, 5), kappa.prior = "uniform",
                        kappa.par = c(0, 5), type = "jackson")
```

3 Fitting NMA-regression models

Here we use the Stroke dataset which is discussed in Section 4.3. There are four different covariates available. We only use `age` covariate to fit a NMA-regression model as is done in the main text. Firstly, we delete the study in which `age` covariate information is not available. Then, centered covariate information can be given by `covariate = 'age'` as follows:

```
data("Strokedat", package = "nmaINLA")
# deleting 13th study
Strokedat.mreg <- Strokedat[-c(13),]
# centering the covariate
Strokedat.mreg$age <- Strokedat.mreg$age - mean(Strokedat.mreg$age)
```

```r
# data preparation for INLA
StrokedatINLA.mreg <- create_INLA_dat(dat = Strokedat.mreg,
                                      armVars = c('treatment' = 't','responders' = 'r',
                                                  'sampleSize' = 'n'),
                                      nArmsVar = 'na',
                                      design = 'des',
                                      covariate = 'age')
```

Then, a Consistency NMA-regression model can be fitted by specifying `mreg = TRUE`:

```r
fit.Stroke.CONS.MREG.INLA <- nma_inla(StrokedatINLA.mreg, likelihood = "binomial",
                                      fixed.par = c(0, 1000), tau.prior = "uniform",
                                      tau.par = c(0, 2), type = 'consistency',
                                      mreg = TRUE)
```

Feedback and comments on `nmaINLA` are always welcome.
Bug reports can be sent to https://github.com/gunhanb/nmaINLA/issues.

4 R version and packages used to generate this document

R version: R version 3.4.0 (2017-04-21)
Base packages: stats, graphics, grDevices, utils, datasets, methods, base
Other packages: nmaINLA, INLA, Matrix, sp, knitr
Versions of other packages (respectively): 0.1.1, 0.0.1485844051, 1.2.10, 1.2.4, 1.16
This document was generated on Juli 04, 2017 at 23:44.

References

R Core Team (2016). *R: A Language and Environment for Statistical Computing.* R Foundation for Statistical Computing, Vienna, Austria.
URL `https://www.R-project.org/`

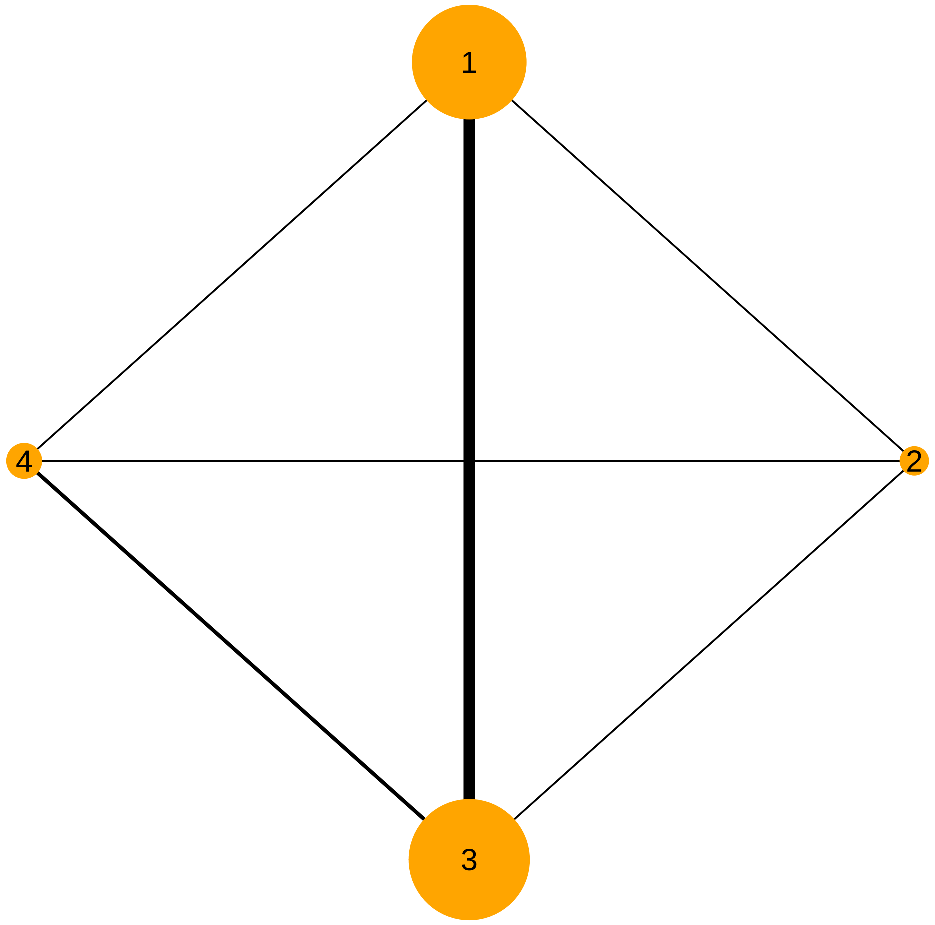

Figure 1: Network of trials of Smoking cessation.

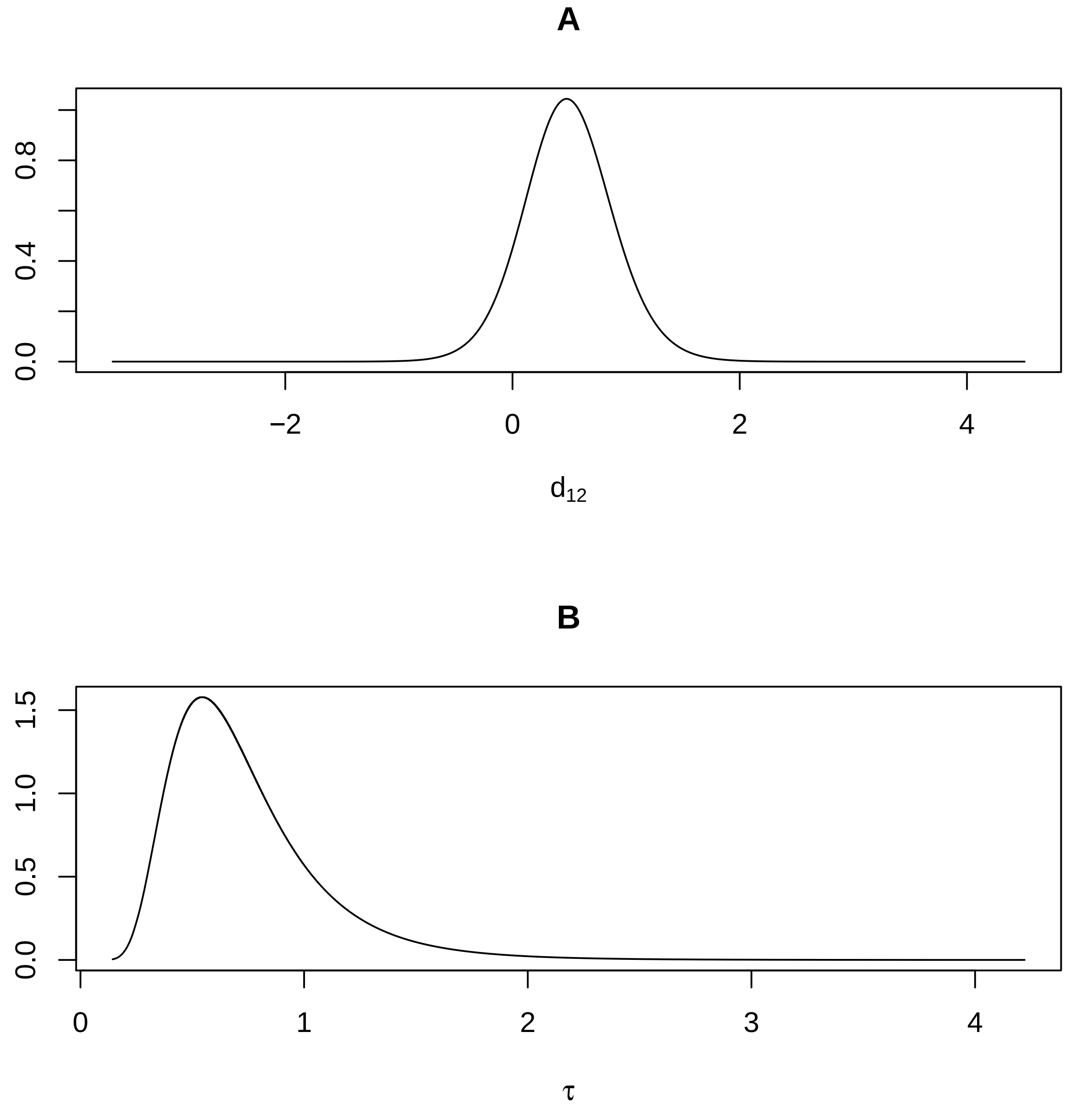

Figure 2: Plot for the marginal posterior density of $d_{1,2}$ and τ^2.
